# A global model for predicting the arrival of imported dengue infections

**DOI:** 10.1371/journal.pone.0225193

**Published:** 2019-12-04

**Authors:** Jessica Liebig, Cassie Jansen, Dean Paini, Lauren Gardner, Raja Jurdak

**Affiliations:** 1 Data61, Commonwealth Scientific and Industrial Research Organisation, Brisbane, Queensland, Australia; 2 Communicable Diseases Branch, Department of Health, Brisbane, Queensland, Australia; 3 Health & Biosecurity, Commonwealth Scientific and Industrial Research Organisation, Canberra, Australian Capital Territory, Australia; 4 Department of Civil Engineering, Johns Hopkins University, Baltimore, Maryland, United States of America; 5 School of Civil and Environmental Engineering, University of New South Wales, Sydney, New South Wales, Australia; 6 School of Electrical Engineering and Computer Science, Queensland University of Technology, Brisbane, Queensland, Australia; 7 School of Computer Science and Engineering, University of New South Wales, Sydney, New South Wales, Australia; CEA, FRANCE

## Abstract

With approximately half of the world’s population at risk of contracting dengue, this mosquito-borne disease is of global concern. International travellers significantly contribute to dengue’s rapid and large-scale spread by importing the disease from endemic into non-endemic countries. To prevent future outbreaks and dengue from establishing in non-endemic countries, knowledge about the arrival time and location of infected travellers is crucial. We propose a network model that predicts the monthly number of dengue-infected air passengers arriving at any given airport. We consider international air travel volumes to construct weighted networks, representing passenger flows between airports. We further calculate the probability of passengers, who travel through the international air transport network, being infected with dengue. The probability of being infected depends on the destination, duration and timing of travel. Our findings shed light onto dengue importation routes and reveal country-specific reporting rates that have been until now largely unknown. This paper provides important new knowledge about the spreading dynamics of dengue that is highly beneficial for public health authorities to strategically allocate the often limited resources to more efficiently prevent the spread of dengue.

## Introduction

The well connected structure of the global air transportation network and the steadily increasing volume of international travel has a vast impact on the rapid, large-scale spread of arboviral and other diseases [1–7]. A recent example of disease introduction to a novel region is the spread of the Zika virus from Brazil to Europe, the United States and other countries, which prompted the World Health Organisation (WHO) to announce a public health emergency of international concern in early 2016. Investigations confirmed that international viraemic travellers were a major contributing factor to the rapid spread [8].

With an estimated 50-100 million symptomatic infections each year [9, 10], dengue is ranked the most important mosquito-borne disease [11, 12]. The rapid geographic spread is, to a great extent, driven by the increase in international air travel [13, 14]. In addition, dengue is severely under-reported, making it extremely challenging to monitor and prevent the spread of the disease. Presumably, 92% of symptomatic infections are not reported to health authorities [10]. Low reporting rates can have many reasons, including low awareness levels and misdiagnosis [9, 15].

Due to the rapid global spread of dengue as well as severe under-reporting, many countries are facing the threat of ongoing local transmission in the near future [11]. In non-endemic countries, local outbreaks are usually triggered by an imported case [16], a person who acquired the disease overseas and transmitted the virus to local mosquitoes. To prevent ongoing dengue transmission in non-endemic countries, it is critical to forecast the importation of disease cases into these areas and move from responsive containment of dengue outbreaks to proactive outbreak mitigation measures.

The majority of existing models forecast relative rather than absolute risk of dengue importation and are unable to predict the total number of imported disease cases [13, 17, 18]. The few models that can predict absolute numbers are region-specific rather than global [19–21]. The most recently proposed model estimates the total number of imported dengue cases for 27 European countries [21], however, the model has several limitations: (i) Monthly incidence rates were based on dengue cases reported to the World Health Organisation (WHO) despite dengue being under-reported and the general consensus that the actual number of cases is much higher than the figures published by the WHO [9, 10]; (ii) Only 16 countries were considered as possible sources of importation. The authors reason that these 16 countries contribute 95% of all global dengue cases, referring to numbers published by the WHO. Since African countries do not report to the WHO, and dengue remains an under-reported disease in many other countries [22–25], it is likely that the percentage contribution to the number of global dengue cases by the 16 selected countries is strongly biased; (iii) Seasonal distributions of dengue cases were inferred based on information from only two source countries (Latin American countries were assumed to have similar seasonalities to Brazil, while Thailand served as a proxy for countries in South-East Asia). The assertion that all countries within a given global region experience similar seasonal fluctuations in dengue infections is likely inaccurate. For example, dengue notifications peak between April and December in Thailand, while Indonesia reports the highest number of dengue cases from November to April [26].

The contribution of this paper is twofold. First, we develop a network model that overcomes the limitations of previous models by employing global air passenger volumes, country-specific dengue incidence rates and country-specific temporal infection patterns. We construct weighted directed networks, using data collected by the International Air Transportation Association (IATA) to capture the movement of air passengers. We calculate monthly, country-specific dengue incidence rates by combining data from the Global Health Data Exchange [27], the most comprehensive health database, and known seasonal patterns in reported dengue infections [26]. Further, we distinguish between two categories of travellers: returning residents and visitors. The number of days people from these two categories spend in an endemic country, and therefore the risk of being infectious on arrival, vary greatly. The model predicts the number of imported dengue cases per month for any given airport and can be applied with relative ease to other vector-borne diseases of global concern, such as malaria, Zika or chikungunya.

Second, we apply the model to infer time-varying, region-specific reporting rates, defined as the ratio of reported to actual infections. Dengue reporting rates vary greatly across space and time, often by several orders of magnitude, and hence are difficult to determine [10]. The usual approach towards estimating country-specific reporting rates is to carry out cohort or capture-recapture studies that can be costly, are time consuming and may be biased [28]. Consequently, dengue reporting-rates remain unknown for most countries [10].

In this paper we focus on those countries that are most at risk of dengue introduction, i.e. non-endemic countries with vector presence. These countries will have the greatest benefit from our model as knowledge about the likely arrival times and places of infected people is crucial to prevent local outbreaks.

## Materials and methods

CSIRO’s human research ethics committee CSSHREC has approved this study (approval number: Ethics Clearance 142/16). All data were analysed anonymously and individuals cannot be identified.

### IATA data

The International Air Transportation Association (IATA) has approximately 280 airline members who together contribute to approximately 83% of all air traffic. Data is collected in form of travel routes, detailing the origin, destination and stopover airports. It contains over 10,000 airports in 227 different countries and dependencies. For each route the total number of passengers per month is given. We do not have any information on stopover times and whether passengers are leaving the airport during their stopover and therefore assume that all passengers continue their journey to the final destination instantly. Table A in S1 File lists the IATA 3-Letter Codes used to abbreviate airports in the main manuscript. As the recorded itineraries do not include any travel on chartered flights, we compare the IATA passenger volumes to official airport passenger statistics [29–47] to quantify the potential discrepancies between actual travel patterns and that reported by IATA. Table B in S1 File lists the countries where the difference in passenger numbers is greater than 15% (at country level) and countries where airport statistics were not available and the tourist data suggests inaccuracies in the IATA data (i.e. the number of tourists arriving in a particular country is larger than the total number of passengers arriving). We also excluded Singapore as a source of importation for Australia for the following reason: The Department of Home Affairs publishes Arrival Card data [48] that can be used to validate the IATA data. A comparison of the monthly travel volume from Singapore to Australia revealed that the IATA data overestimates travel volumes by approximately 112% on average in 2011 and 2015. This may be due to individuals who travel from other countries to Singapore and then directly continue to Australia and do not book their entire trip in one itinerary (this would be recorded as two separate trips in the IATA data that cannot be linked to each other). Due to this large discrepancy in the travel data we believe that our model will significantly overestimate the number of dengue infections imported from Singapore, and therefore exclude it as a source country for Australia.

### The air transportation network

We begin by constructing twelve weighted, directed networks, using IATA data, to represent the monthly movement of air passengers during a given year. The networks are denoted $G_{m}=\left(V,E\right)$, with *m* = 1,…,12 indicating the month of the year. The node set *V* comprises more than 10,000 airports recorded by IATA. To distinguish the travellers by their country of embarkation, we represent the edges of the network as ordered triples, (*i*, *j*, *ω*_*i*,*j*_(*c*, *k*)) ∈ *E*, where *i*, *j* ∈ *V* and *ω*_*i*,*j*_(*c*, *k*) is a function that outputs the number of passengers who initially embarked in country *c* with final destination airport *k* and travel from airport *i* to airport *j* as part of their journey.

### Incidence rates and seasonal distributions

Calculating the number of infected passengers requires daily infection probabilities. We derive these from country-level yearly estimates of symptomatic dengue incidence rates that are published together with their 95% confidence intervals by the Global Health Data Exchange [27]. The estimates are obtained using the model published in [10] and account for under-reporting.

We first deduce monthly incidence rates using information on dengue seasonality published by the International Association for Medical Assistance to Travellers [26]. To do so we associate a weight with each month that indicates the intensity of transmission. To assign the weights we use a modified cosine function with altered period that matches the length of the peak-transmission season. The function is shifted and its amplitude adjusted so that its maximum occurs midway through the peak-season with value equal to the length of the peak-season divided by 2*π*. The months outside the peak-season receive a weight of one if dengue transmission occurs year around and a weight of zero if dengue transmission ceases outside the peak-season. The weights are then normalised and multiplied by the yearly incidence rate for the corresponding country. Normalising the weights ensures that the sum of the monthly incidence rates is equal to the yearly incidence rate. To calculate the lower and upper bounds of the monthly incidence rates, we multiply the normalised weights by the lower and upper bounds of the 95% confidence interval given for the yearly incidence rates.

The average probability, *β*_*c*,*m*_, of a person becoming infected on any given day during month *m* in country *c* is then given by

$$\begin{array}{c} \beta_{c,m}=1-e^{-\gamma_{c,m}/d_{m}}, \end{array}$$ (1)

where *γ*_*c*,*m*_ is the monthly dengue incidence rate in country *c* during month *m* and *d*_*m*_ is number of days in month *m*. Note that Eq (1) converts the daily incidence rate into the probability of a single person becoming infected with dengue on any given day during month *m*.

### Inferring the number of infected passengers

Next, we present a mathematical model that approximates the number of dengue-infected people for each edge in the network $G_{m}\left(V,E\right)$. The time between being bitten by an infectious mosquito and the onset of symptoms is called the intrinsic incubation period (IIP). This period closely aligns with the latent period, after which dengue can be transmitted to mosquitoes [49]. The IIP lasts between 3 and 14 days (on average 5.5 days) and was shown to follow a gamma distribution of shape 53.8 and scale equal to 0.1 [50]. After completion of the IIP a person is infectious for approximately 2 to 10 days (on average 5 days) [50, 51]. The length of the infectious period was shown to follow a gamma distribution of shape 25 and scale equal to 0.2 [50]. We denote the sum of the IIP and the infectious period by *n*, which is rounded to the nearest integer after the summation. For travellers to import the infection from country *c* into a new location *r* they must have been infected with dengue within the last *n* − 1 days of their stay in country *c*. We now consider the following two cases: *t*_*c*_ ≥ *n* − 1 and *t*_*c*_ < *n* − 1, where *t*_*c*_ is number of days spent in country *c* before arriving in region *r*. Since we do not know the exact date of arrival for travellers, we assume that arrival and departure dates fall within the same month and hence *β*_*c*,*m*_ is the same for every day during the travel period.

If *t*_*c*_ ≥ *n* − 1, that is the individual spent more time in country *c* than the sum of the lengths of the IIP and the infectious period, the probability of not being infected on return is equal to ${\left(1-\beta_{c,m}\right)}^{t_{c}}+[1-{\left(1-\beta_{c,m}\right)}^{t_{c}-\left(n-1\right)}]$. The first term covers the possibility that the individual did not get infected whilst staying in country *c* and the second term covers the possibility that the individual got infected and recovered before arriving at a given airport (see Fig A in S1 File). Hence, the probability of a person, who arrives at a given airport from country *c* during month *m*, being infected with dengue is given by

$$\begin{array}{ccc} p_{c,m} & = & 1-[{\left(1-\beta_{c,m}\right)}^{t_{c}}+1-{\left(1-\beta_{c,m}\right)}^{t_{c}-\left(n-1\right)}]\\ & = & {\left(1-\beta_{c,m}\right)}^{t_{c}-\left(n-1\right)}-{\left(1-\beta_{c,m}\right)}^{t_{c}}. \end{array}$$ (2)

If *t*_*c*_ < *n* − 1, that is the individual spent less time in country *c* than the sum of the lengths of the IIP and the infectious period, the probability of not being infected on return is equal to ${\left(1-\beta_{c,m}\right)}^{t_{c}}$, which covers the possibility that the individual did not get infected whilst staying in country *c*. Since *t*_*c*_ < *n* − 1, the probability of recovery before arriving at a given airport is zero. Hence, the probability of a person, who arrives from country *c* at a given airport during month *m*, being infected with dengue is given by

$$\begin{array}{c} p_{c,m}=1-{\left(1-\beta_{c,m}\right)}^{t_{c}}. \end{array}$$ (3)

We distinguish between two different types of travellers arriving at a given airport of region *r*: returning residents and visitors. We define a returning resident as a traveller who resides in region *r* and a visitor as a traveller who resides in country *c* and visits region *r*. Returning residents are expected to have stayed a couple of weeks in the endemic country, while visitors may have spent their whole life in the country.

Since we lack information on how long each individual spent in country *c* before arriving at an airport of region *r*, we substitute parameter *t*_*c*_ by ${\langle t\rangle }_{c}^{res}$ if the person is a returning resident, ${\langle t\rangle }_{c}^{res}$ being the average number of days a returning resident spends in country *c* before returning home. If the person is a visitor, parameter *t*_*c*_ is substituted by ${\langle t\rangle }_{c}^{\text{vis}}$, the average number of days a visitor spends in country *c* before arriving at an airport of region *r*. We distinguish between returning residents and visitors since ${\langle t\rangle }_{c}^{\text{res}}⪡{\langle t\rangle }_{c}^{\text{vis}}$.

We assume that the length of stay for returning residents follows a normal distribution with mean equal to 15 days and standard deviation of 2, i.e. ${\langle t\rangle }_{c}^{res}∼N\left(15,2\right)$. A previous study has shown that employees around the world are on average entitled to approximately 15 days of annual leave [52]. On the other hand, visitors likely spent all their lives in the endemic country. We assume that ${\langle t\rangle }_{c}^{vis}∼N\left(\mu_{vis},0.1\mu_{vis}\right)$, where *μ*_*vis*_ is equal to *c*’s median population age. Median population ages by country are published in the World Factbook by the Central Intelligence Agency [53].

For simplicity we do not take immunity to the different dengue strains into consideration.

### Proportion of returning residents and visitors

Lastly, we need to infer the proportions of returning residents and visitors. As this information is not contained in the IATA itineraries, we use international tourism arrival data from the World Tourism Organisation [54]. The data contains the yearly number of international tourist arrivals by air for each destination country. From the IATA data we calculate the total number of arrivals per year for each country and hence can infer the ratio of visitors to returning residents. As we lack sufficient data, we assume that the ratio of visitors to residents is the same for each month.

### Calculating the absolute number of infected passengers

Given the above, we can now determine the number of infected passengers *I*_*k*,*m*_ arriving at airport *k* during month *m* as follows:

$$\begin{array}{c} I_{k,m}=\sum_{i,j,c}\omega_{i,j}\left(c,k\right)[qp_{c,m}^{res}+\left(1-q\right)p_{c,m}^{vis}], \end{array}$$ (4)

where *q* is the proportion of residents inferred from the international tourism arrival data,

$$\begin{array}{c} p_{c,m}^{res}=\{\begin{array}{cc} {\left(1-\beta_{c,m}\right)}^{{\left\langle t\right\rangle }_{c}^{res}-\left(n-1\right)}-{\left(1-\beta_{c,m}\right)}^{{\left\langle t\right\rangle }_{c}^{res}} & {\left\langle t\right\rangle }_{c}^{res}\geq n-1\\ 1-{\left(1-\beta_{c,m}\right)}^{{\left\langle t\right\rangle }_{c}^{res}} & {\left\langle t\right\rangle }_{c}^{res}<n-1, \end{array} \end{array}$$ (5)

and

$$\begin{array}{c} p_{c,m}^{vis}=\{\begin{array}{cc} {\left(1-\beta_{c,m}\right)}^{{\left\langle t\right\rangle }_{c}^{vis}-\left(n-1\right)}-{\left(1-\beta_{c,m}\right)}^{{\left\langle t\right\rangle }_{c}^{vis}} & {\left\langle t\right\rangle }_{c}^{vis}\geq n-1\\ 1-{\left(1-\beta_{c,m}\right)}^{{\left\langle t\right\rangle }_{c}^{vis}} & {\left\langle t\right\rangle }_{c}^{vis}<n-1. \end{array} \end{array}$$ (6)

### Evaluation of the model’s uncertainty

We performed a thousand runs of the model for each edge in the network, drawing the parameters from their respective distributions, to calculate the mean and standard deviation of dengue-infected passengers. In addition, we have conducted a global sensitivity analysis to identify the model parameters with the greatest influence. We used Sobol’s method [55] with 100,000 samples to carry out the sensitivity analysis. The parameter ranges are shown in Table 1. The analysis was done with SALib [56], an open-source Python library.

**Table 1:** The model parameter ranges used in Sobol’s method.

Parameter	Range
*β*_*c*,*m*_	[0.000001, 0.000445]
*t*_*c*_ (days)	[1, 29200]
*n* (days)	[5, 24]

## Results

**Fig 1:**
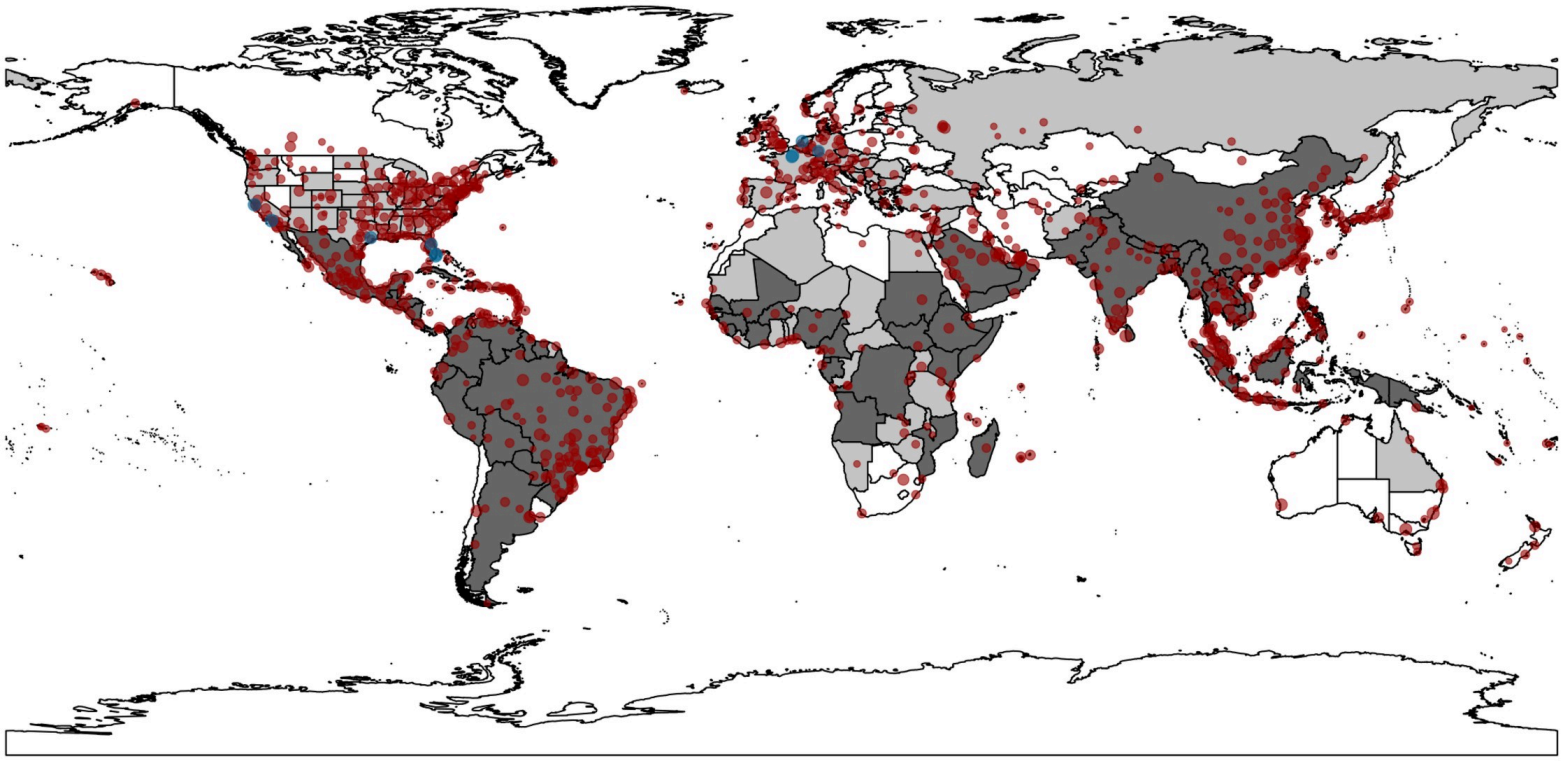
Predicted dengue importations for August 2015. The map shows the output of our model for August 2015.The area of a node increases with the number of dengue cases imported through the corresponding airport. Airports that are predicted to not receive any infections are not shown on the map. Endemic countries are coloured dark grey. Countries that are non-endemic and where dengue vectors *Aedes aegypti* and/or *Aedes albopictus* are present are coloured in light grey. The blue circles correspond to the top ten airports identified in Fig 2. The map was created with the Python GeoPandas package and publicly available shapefiles from Natural Earth (http://www.naturalearthdata.com/).

We run our model for two different years to explore the robustness of the proposed methodology. Specifically, the analysis is conducted for 2011 and 2015. The results for the year 2015 are presented in the main manuscript, while the results for 2011 are presented in the supplementary material. Fig 1 shows the number of predicted imported dengue infections per airport for August 2015, where the area of a node increases with the number of dengue cases imported through the corresponding airport. The map clearly shows that many non-endemic regions where the dengue-transmitting vectors *Aedes aegypti* or *Aedes albopictus* are present (coloured in light grey) have airports that are predicted to receive a high number of dengue infections. For a list of dengue endemic and non-endemic countries see Table C in S1 File. As resources for the control and prevention of dengue are often limited [57], these countries face a high risk of future endemicity.

**Fig 2:**
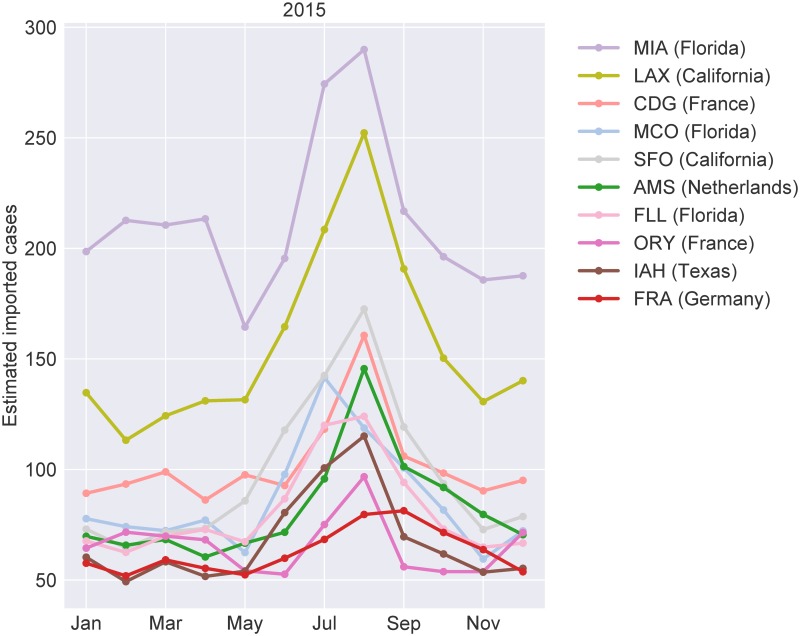
Predicted monthly dengue importations by airport for 2015. The number of predicted imported dengue infections for the top ten airports in non-endemic countries/states with vector presence for each month in 2015. A break in a line indicates that the corresponding airport was not amongst the top ten during the respective month. Airports are abbreviated using the corresponding IATA code. A full list of abbreviations can be found in the supplementary material (see Table A in S1 File).

In Fig 2 and Fig B in S1 File we plot the number of predicted dengue importations over time for the ten airports that receive the highest number of cases, lie in non-endemic regions with vector presence and where local cases have been reported in the past (more detailed plots with confidence intervals are shown in Fig C in S1 File). While the majority of airports listed in Fig 2 and Fig B in S1 File are predicted to receive between 50 and 150 cases each month, Miami International Airport (MIA) is estimated to receive between 146 and 309 cases each month during both years. With Orlando International Airport (MCO) and Fort Lauderdale–Hollywood International Airport (FLL) also represented amongst the airports with the highest number of imported cases, Florida faces a high risk of local dengue outbreaks. Los Angeles International Airport (LAX) is predicted to receive the second highest number of imported cases. In 2011 its monthly predictions vary between 97 and 205 cases and in 2015 between 113 and 253 cases. The remaining airports listed in Fig 2 and Fig B in S1 File are located in France, Germany, the Netherlands, Texas, and Queensland, Australia. A full ranking of all airports located in non-endemic countries with vector presence can be found in Table D in S1 File.

In addition to calculating the number of imported dengue infections per airport, the model further provides the number of infected passengers travelling between any two airports, thus revealing common importation routes. Table 2 and Table E in S1 File list the routes that carry the highest number of infected passengers whose final destinations lie in non-endemic countries with vector presence. Table F in S1 File lists the routes that carry the highest number of infected passengers whose final destinations lie in non-endemic countries irrespective of whether vectors are present. For example, the route between Denpasar and Perth is ranked third in 2011 in Table F in S1 File, but it is not considered in the ranking shown in Table E in S1 File, as there are no vectors in Perth. Fig D in S1 File shows a map of all importation routes into non-endemic countries with vector presence.

**Table 2:** The ten routes with the highest predicted number of dengue-infected passengers with final destinations in non-endemic countries with vector presence.

Orig.	Dest.	Pax	Month
SJU (Puerto Rico)	MCO (Florida)	51	Jul
PTP (Guadeloupe)	ORY (France)	37	Aug
FDF (Martinique)	ORY (France)	34	Aug
SJU (Puerto Rico)	FLL (Florida)	32	Jul
TPE (Taiwan)	LAX (California)	31	Aug
GRU (Brazil)	MIA (Florida)	29	Apr
DEL (India)	KBL (Afghanistan)	27	Aug
GDL (Mexico)	LAX (California)	24	Aug
CUN (Mexico)	MIA (Florida)	24	Aug
CUN (Mexico)	LAX (California)	22	Aug

The table lists the direct routes with the highest predicted volume of dengue-infected passengers who continue to travel to non-endemic regions with vector presence and where local outbreaks have been reported in the past. The last column records the month during which the highest number of infected passengers are predicted.

In both years the highest predicted number of infected passengers are recorded during the northern hemisphere’s summer. The route between São Paulo International Airport (GRU) and Miami International Airport (MIA) is the exception, where the highest number of infected passengers is predicted during April. The routes with the highest estimated number of dengue-infected passengers terminate at airports in countries that are non-endemic and where dengue-transmitting vectors are present.

### Returning residents and visitors

Next, we aggregate airports by country/state to predict the number of imported dengue infections on a coarser level. For non-endemic countries that cover an area larger than 5,000,000 km^2^ and where dengue vectors are present we aggregate airports by state. These countries are Russia, the United States of America and Australia. The comparison between passenger volumes recorded by IATA and official airport statistics indicated that the IATA data for Russia may be inaccurate, i.e. the difference in passenger numbers is larger than 15% (see Material and Methods). Hence, we did not perform a state-level analysis for this country. In Australia vectors are present only in Queensland [58]. While vectors have been observed in more than 40 different US states, autochthonous cases have been reported only in California, Florida, Hawaii and Texas [59].

**Fig 3:**
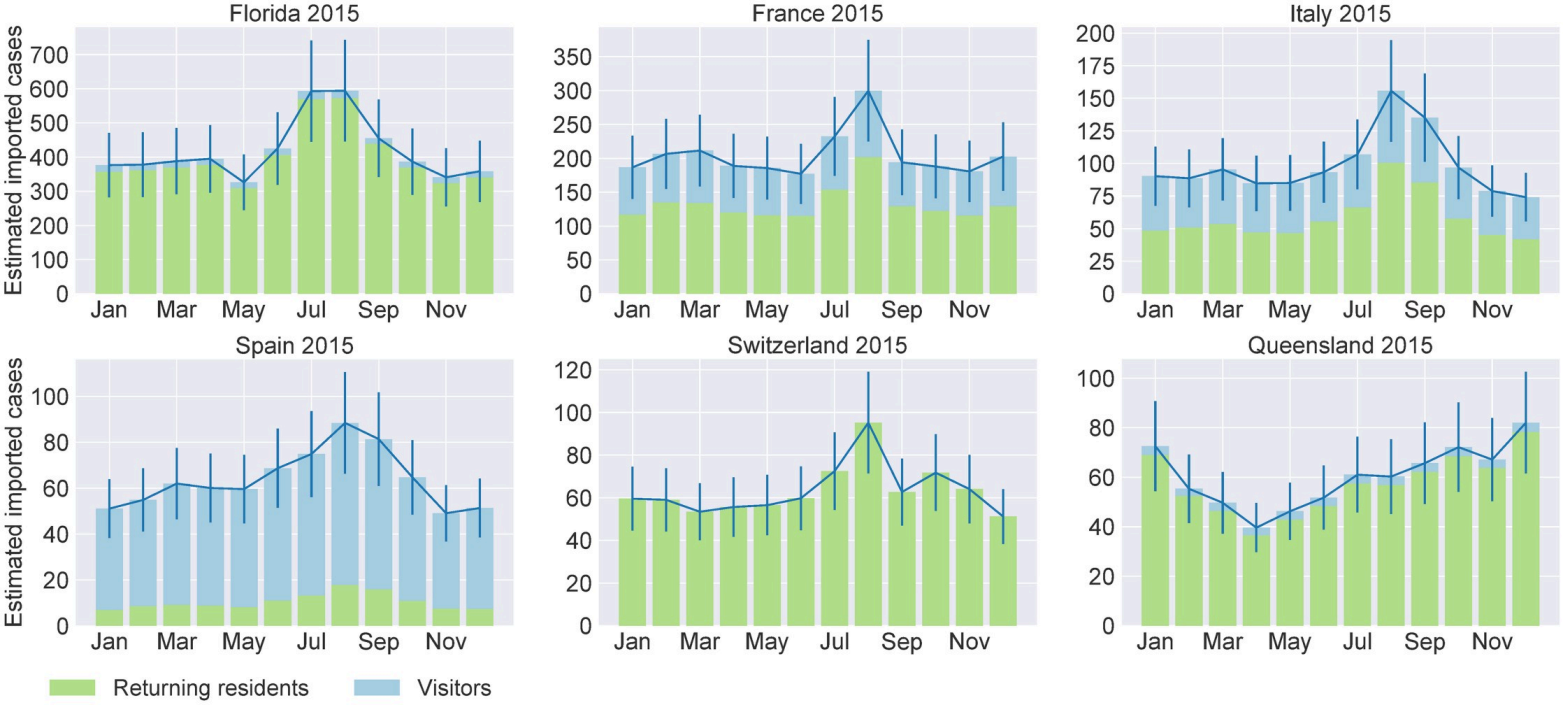
Predicted dengue infections imported by returning residents and visitors in 2015. Here we show the results for non-endemic countries/states with vector presence with the highest number of predicted imported dengue cases in 2015. The bars are stacked to distinguish between returning residents (green) and visitors (blue). The blue solid line corresponds to the total number of imported cases. The error bars correspond to the model’s coefficient of variation (see Material and methods). The six countries were selected because they are predicted to receive the highest number of dengue importations, are non-endemic and dengue vectors are established.

Our model separately calculates the number of dengue-infected people amongst returning residents and visitors and hence we can identify which of these groups is more likely to import the disease into a given country or state. Fig 3 and Fig E in S1 File show the results for six non-endemic countries/states with vector presence that are predicted to receive the highest number of dengue importations each month. Results for the remaining countries and states are shown in Figs F–K in S1 File. We observe that the contributions of returning residents and visitors to the total number of imported dengue infections is predicted to vary greatly between the different countries and states. In Florida and Queensland returning residents are predicted to be the main source of dengue importation. In France and Italy approximately one third of all dengue infections are predicted to be imported by visitors while in Spain visitors import around 75% of all imported cases. For Switzerland we do not have any information about the ratio of returning residents to visitors. For the United States there is evidence in the form of surveillance reports that returning residents are indeed the main contributors to dengue importations [60]. For Queensland we predict that 95% and 94% of infections were imported by returning residents in 2011 and 2015, respectively. Our predictions are supported by Queensland’s dengue notification data (provided by Queensland Health), showing that 97% and 92% of all dengue importations in 2011 and 2015, respectively, were imported by returning residents.

### Countries of acquisition

**Fig 4:**
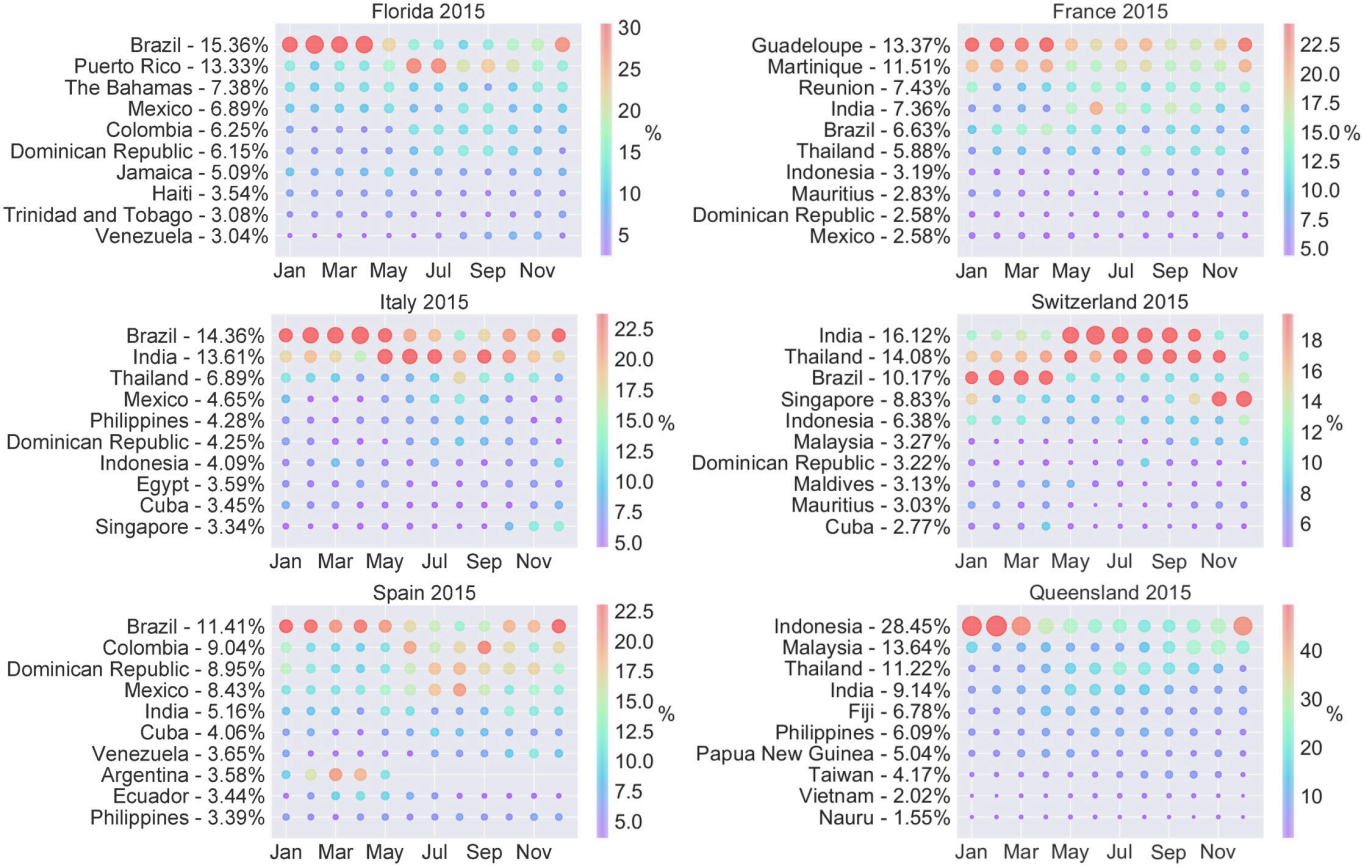
Predicted percentage contribution of dengue importations by country of acquisition in 2015. The predicted percentage contribution by source country and month in 2015. The size and colour of the circles indicate the percentage contribution of the corresponding country to the total number of imported cases. The *y*-labels indicate the yearly percentage contribution of the corresponding source country.

In addition to being able to distinguish between returning residents and visitors, the model also divides the imported cases according to their places of acquisition. Fig 4 and Fig L in S1 File show the model’s estimated percentage contribution of dengue importations by source country.

Florida is predicted to import most infections from the Caribbean and Latin America, with infections acquired in Puerto Rico (PRI) predicted to peak during June and July and infections acquired in Brazil predicted to peak between January and April. We hypothesise that Florida receives such a high number of imported dengue cases due to its close proximity to the Caribbean, which has been endemic since the 1970s [61]. France is predicted to receive many infections from the Caribbean, in particular from Martinique and Guadeloupe which are French overseas regions and hence a high volume of air traffic from these regions to metropolitan France is expected. These predictions align with the fact that outbreaks of dengue in France coincide with outbreaks in the French West Indies, where most reported cases are acquired [62, 63]. In Italy the model predicts that the most common countries of acquisition are India and Brazil. India and Brazil are also the most common countries of acquisition for Switzerland in 2011. In 2015 Switzerland is predicted to receive most of their dengue importations from India and Thailand. Spain is predicted to import the majority of infections from Latin America and the Caribbean. For Queensland the model predicts that imported cases are acquired mostly in South-East Asia with Indonesia being the largest source. This is in agreement with previous studies [64] and the dengue case data that was provided by Queensland Health. In addition, we performed a rank-based validation of these results.

**Fig 5:**
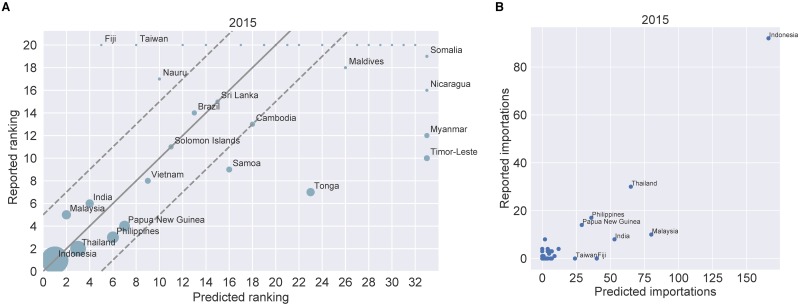
Rank-based validation and correlation between reported and predicted imported cases for Queensland in 2015. **(A)** Countries are ranked by the total number of predicted and reported imported dengue cases. The reported ranking is then plotted against the predicted ranking. Countries that were ranked by the model, but did not appear in the dataset receive a rank of *i* + 1, were *i* is the number of unique importation sources according to the dengue case data. Similarly, countries that appeared in the data and were not ranked by the model receive a rank of *i* + 1. For circles that lie on the *x* = *y* line (grey solid line) the predicted and reported rankings are equal. Circles that lie between the two dashed lines correspond to countries with a difference in ranking that is less than or equal to five. The circle areas are scaled proportionally to the number of reported cases that were imported from the corresponding country. Spearman’s rank correlation coefficient between the absolute numbers of reported and predicted importations is equal to 0.6. **(B)** The absolute number of reported dengue importations are plotted against the absolute number of predicted importations.

We obtained dengue case data from Queensland Health, which records the places of acquisition for each dengue case reported in Queensland. We rank the countries of acquisition by the total number of predicted and reported dengue-infected people who arrive in Queensland. We then plot the reported ranking against the predicted ranking. In addition, we plot the absolute number of reported importations against the absolute number of predicted importations and calculate Spearman’s rank correlation coefficient. Fig 5 and Fig M in S1 File show the results.

The rank-based validation of our model demonstrates that overall, the model captures the different importation sources well. It does particularly well for the countries from which Queensland receives the most infections. Spearman’s rank correlation coefficient is equal to 0.6 for the year 2015 and equal to 0.58 for the year 2011. Below we explain some of the differences between the data and the model output.

For the rank-based validation the two largest outliers in both years are Fiji and Taiwan. The predicted ranking for Fiji in 2011 is 2, while the reported ranking is 10. In 2015 we estimate Fiji to be ranked fifth, however no cases were reported in 2015 and hence Fiji is ranked last amongst the reported cases. According to the Fijian government tourists are less likely to contract the disease than local residents as they tend to stay in areas that are not infested by *Aedes aegypti* mosquitoes [65] or where there is likely considerable control effort undertaken by tourism accommodation operators. Since the incidence rates incorporated into our model do not distinguish between different regions of a source country, the model is unable to account for such nuances. In 2011 and 2015 we estimate Taiwan to be ranked seventh and eighth, respectively, however no cases were reported in both years. This result is surprising as dengue occurs year-round in Taiwan [26] and approximately 44,000 and 16,000 Queensland residents travelled to Taiwan in 2011 and 2015, respectively.

Some of the differences between the observed percentages and the predicted percentages can be explained by under-reporting. It is possible that dengue awareness among travellers to one country is greater than the awareness amongst travellers to another country. Travellers with higher awareness levels are more likely to report to a doctor if feeling unwell after their return.

### Country-specific reporting rates

The reporting rate of a disease is defined as the ratio of reported infections to actual infections. Dengue reporting rates vary greatly across space and time and are difficult to determine [10]. The usual approach to estimating country-specific reporting rates is to carry out cohort or capture-recapture studies that can be costly, are time consuming and may be biased [28].

We utilised our model to infer country- and state-specific reporting rates of imported cases by performing a least squares linear regression without intercept.

Table 3 and Table G in S1 File show the estimated yearly and seasonal reporting rates of imported cases for Queensland, Florida, France, Italy and Spain. To distinguish locally acquired and imported cases in Queensland, we use case-based data from Queensland Health where the country of acquisition is recorded. Travel-related dengue cases reported in Europe are published by the European Centre for Disease Prevention and Control (http://ghdx.healthdata.org/gbd-results-tool). Data for Florida is available from the Florida Department of Health (http://www.floridahealth.gov/diseases-and-conditions/mosquito-borne-diseases/surveillance.html).

**Table 3:** Yearly and seasonal reporting rates of imported cases in 2015.

	Dec-Feb	Mar-May	Jun-Aug	Sep-Nov	Yearly
Queensland	32.4	48.9	18.6	22.6	28.6
Spain	14	14	31.7	26.3	23.5
Italy	4.5	6.8	9.2	13.1	9
France	3.8	6.9	9.7	7.1	7.2
Florida	0.9	0.7	1.2	2.7	1.4

The table shows the estimated reporting rates of imported cases for Queensland, Spain, Italy, France and Florida. We estimate the reporting rates by using a least squares linear regression without intercept.

The results show that estimated reporting rates of imported cases are highest in Queensland, in particular during autumn. This is expected as dengue awareness campaigns are intensified between November and April [66]. In contrast, Florida has the lowest dengue reporting rate (1.3% in 2011 and 1.4% in 2015). This finding is supported by a previous study which found that awareness levels in Florida are extremely low [67]. The estimated reporting rates for the European countries are also low; however, the model predicts a substantial increase from 2011 to 2015. The question why reporting rates in Queensland are higher is challenging to answer, as we do not have any information about the true number of imported cases. However, Queensland has one of the best dengue prevention programs in the world. According to Queensland Health, other states and countries frequently ask for training and advice regarding surveillance and awareness campaigns.

### Model uncertainty

We found that the average coefficient of variation of our importation model is 19.5% across both years. That is, the model’s standard deviation is on average equal to 19.5% of its mean. Fig N in S1 File shows the distribution of the coefficient of variation for several destinations.

The results from the global sensitivity analysis show that *t*_*c*_ is the most important of the three model parameters with a total-order index of 0.94 (see Fig O in S1 File). The different values of the first-order and total-order indices indicate interaction between the model parameters. The second-order indices show that there is significant interaction between parameters *t*_*c*_ and *β*_*c*,*m*_ with a second-order index of 0.19, as well as between parameters *t*_*c*_ and *n* with a second-order index of 0.1.

Since the range of parameter *t*_*c*_ is large ([1, 29200] days), we performed the sensitivity analysis again for a shorter range of values ([1, 30] days) that is more realistic for returning residents who spend their holidays in an endemic country. In this case, parameter *β*_*c*,*m*_, with a total-order index of 0.6, is more important than *t*_*c*_, which has a total-order index of 0.35 (see Fig O in S1 File). The second-order indices show that there is still significant interaction between parameters *t*_*c*_ and *β*_*c*,*m*_ with a second-order index of 0.06, and between parameters *t*_*c*_ and *n* with a second-order index of 0.07.

## Discussion

To mitigate the risk of outbreaks from importation of dengue into non-endemic regions it is critical to predict the arrival time and location of infected individuals. We modelled the number of dengue infections arriving each month at any given airport, which enabled us to estimate the number of infections that are imported into different countries and states each month. In addition, the model determines the countries of acquisition and hence is able to uncover the routes along which dengue is most likely imported. Our results can also be used to estimate country- and state-specific reporting rates of imported cases.

Such knowledge can inform surveillance, education and risk mitigation campaigns to better target travellers along high risk importation routes at the most appropriate times. It will also help authorities to more efficiently surveil those airports with the highest risk of receiving dengue-infected passengers.

The model proposed here overcomes many of the shortcomings of previous models, however, it is not without limitations. Validation through comparison of reported cases to predicted cases is infeasible due to the high degree of under-reporting. However, we demonstrate that the coefficient of variation of the model with 19.5% on average is low (see Material and Methods). A rank-based validation for Queensland confirmed that the different importation sources are accurately predicted.

Incidence rates may vary considerably from region to region within the same country [65] and higher resolution data could improve the model’s predictions, as it would better reflect the export of dengue cases from the individual regions. Region-specific incidence rates can, for instance, be combined with spatial patterns of the visiting frequency of travellers to determine the likelihood of travellers to export dengue out of endemic countries. Additional data on individuals’ travel behaviour may also be beneficial, as it can be analysed to improve the estimation of the average time that a person has spent in a specific country before arriving at a given airport. Our assumption that returning residents and visitors are exposed to the same daily incidence rates is a simplification. Further details on the types of accommodation, for example, resorts vs local housing, could also be used to inform the daily incidence rates, due to variations in vector control. The global sensitivity analysis has revealed that *t*_*c*_, the number of days a traveller has spent in country *c*, is the most important model parameter. Hence, additional data on individuals’ travel behaviour may substantially improve the model. Knowledge about the exact age of visitors who reside in non-endemic countries would also improve the model. Currently, we assume that the age of a visitor is equal to the median age of the population of the country in which the visitor resides. In reality, the age of air passengers may differ from the median age, especially for developing countries.

In temperate regions local conditions may not allow for dengue to be transmitted during the winter months. Thus, even a large number of imported cases during those months would not trigger local outbreaks. Variable seasonality patterns due to El Niño Southern Oscillation can affect the spread of dengue in tropical and subtropical regions. An interesting direction for future research is to combine the here proposed model with knowledge of local conditions and weather phenomena like El Niño Southern Oscillation to evaluate the risk of local outbreaks. In this work we studied dengue importation via air travel. In future, we will also consider other modes of transportation to develop a more comprehensive model.

## Supporting information

S1 File Supplementary material. This file contains all supplementary figures and tables.
(PDF)

S2 File Dengue incidence rates for 2011. (CSV)

S3 File Dengue incidence rates for 2015. (CSV)
