# Supplementary material for: A global model for predicting the arrival of imported dengue infections
Source: PLoS One. 2019 Dec 4;14(12):e0225193. doi: 10.1371/journal.pone.0225193 (PMC6892502; doi:10.1371/journal.pone.0225193)
Supplement: S1 File — This file contains all supplementary figures and tables. (PDF) [file pone.0225193.s001.pdf]

Supplementary material

Jessica Liebig, Cassie Jansen, Dean Paini, Lauren Gardner, Raja Jurdak

IATA data

Table A. List of airport abbreviations

IATA 3-Letter Code	Name	City (Country/State)	IATA 3-Letter Code	Name	City (Country/State)
AEP	Jorge Newbery Airport	Buenos Aires (Argentina)	LAX	Los Angeles International Airport	Los Angeles (California)
BKK	Suvarnabhumi Airport	Bangkok (Thailand)	LHR	Heathrow Airport	London (UK)
BNE	Brisbane Airport	Brisbane (Queensland)	MAD	Adolfo Suárez Madrid-Barajas Airport	Madrid (Spain)
BOM	Chhatrapati Shivaji International Airport	Mumbai (India)	MCO	Orlando International Airport	Orlando (Florida)
CDG	Charles de Gaulle Airport	Paris (France)	MEX	Mexico City International Airport	Mexico City (Mexico)
COK	Cochin International Airport	Kochi (India)	MIA	Miami International Airport	Miami (Florida)
CUN	Cancún International Airport	Cancún (Mexico)	MNL	Ninoy Aquino International Airport	Manila (Philippines)
DEL	Indira Gandhi International Airport	New Delhi (India)	MTY	Monterrey International Airport	Apodaca (Mexico)
DFW	Dallas/Fort Worth International Airport	Dallas (Texas)	MXP	Milan Malpensa Airport	Milan (Italy)
DPS	Ngurah Rai International Airport	Denpasar (Indonesia)	NRT	Narita International Airport	Tokyo (Japan)
DXB	Dubai International Airport	Dubai (UAE)	ORY	Paris Orly Airport	Paris (France)
EZE	Ministro Pistarini International Airport	Buenos Aires (Argentina)	PER	Perth Airport	Perth (Western Australia)
FDF	Martinique Aimé Césaire International Airport	Forte-de-France (Martinique)	PTP	Pointe-à-Pitre International Airport	Pointe-à-Pitre (Guadeloupe)
FLL	Fort LauderdaleHollywood International Airport	Miami (Florida)	PUJ	Punta Cana International Airport	Punta Cana (Dominican Republic)
GDL	Miguel Hidalgo y Costilla Guadalajara International Airport	Guadalajara (Mexico)	SAL	Monseñor Óscar Arnulfo Romero International Airport	San Salvador (El Salvador)
GRU	São Paulo International Airport	São Paulo (Brazil)	SDQ	Las Américas International Airport	Punta Caucedo (Dominican Republic)
ICN	Incheon International Airport	Seoul (South Korea)	SFO	San Francisco International Airport	San Francisco (California)
IAH	George Bush Intercontinental Airport	Houston (Texas)	SJU	Luis Muñoz Marín International Airport	San Juan (Puerto Rico)
JFK	John F. Kennedy International Airport	New York City (New York)	STI	Cibao International Airport	Santiago de los Caballeros (Dominican Republic)
KBL	Hamid Karzai International Airport	Kabul (Afghanistan)	TPE	Taiwan Taoyuan International Airport	Taipei (Taiwan)

Table B. List of non-endemic countries where IATA data is inaccurate

Algeria	Bahrain	Bonaire, Saint Eustatius & Saba	Bulgaria
Central African Republic	Croatia	Egypt	Federated States of Micronesia
Finland	Germany	Guinea-Bissau	Greece
Hungary	Iceland	Iran	Israel
Malawi	Morocco	Netherlands	Russian Federation
Serbia	Slovenia	South Africa	South Korea
Tanzania	Togo	The Gambia	Tunisia
Turkey	Uganda	Ukraine	Zambia

Table C. List of countries indicating whether dengue vectors are present and whether the country is endemic. Information about endemicity was obtained from [1]. Information about vector presence was obtained from [2].

Country	Endemic	Vector presence	Country	Endemic	Vector presence	Country	Endemic	Vector presence
Afghanistan	no	yes	Ghana	no	yes	Pakistan	yes	yes
Albania	no	yes	Gibraltar	no	yes	Palau	yes	yes
Algeria	no	yes	Greece	no	yes	Palestine	no	yes
American Samoa	no	yes	Greenland	no	no	Panama	yes	yes
Andorra	no	no	Grenada	yes	yes	Papua New Guinea	yes	yes
Angola	yes	yes	Guadeloupe	yes	yes	Paraguay	yes	yes
Anguilla	yes	yes	Guam	no	no	Peru	yes	yes
Antarctica	no	no	Guatemala	yes	yes	Philippines	yes	yes
Antigua and Barbuda	yes	yes	Guinea	yes	yes	Poland	no	no
Argentina	yes	yes	Guinea-Bissau	no	yes	Portugal	no	yes
Armenia	no	yes	Guyana	yes	yes	Puerto Rico	yes	yes
Aruba	yes	yes	Haiti	yes	yes	Qatar	no	no
Australia	no	yes	Honduras	yes	yes	Reunion	yes	yes
Austria	no	yes	Hong Kong	yes	yes	Romania	no	yes
Azerbaijan	no	no	Hungary	no	yes	Russian Federation	no	yes
Bahrain	no	no	Iceland	no	no	Rwanda	no	yes
Bangladesh	yes	yes	India	yes	yes	Saint Helena	no	no
Barbados	yes	yes	Indonesia	yes	yes	Saint Kitts and Nevis	yes	yes
Belarus	no	no	Inner Hebrides	no	no	Saint Lucia	yes	yes
Belgium	no	yes	Iran	no	no	Saint Pierre and Miquelon	no	no
Belize	yes	yes	Iraq	no	no	Saint Vincent and the Grenadines	yes	yes
Benin	no	yes	Ireland	no	no	Samoa	no	yes
Bermuda	no	yes	Israel	no	yes	Sao Tome and Principe	no	no
Bhutan	yes	yes	Italy	no	yes	Saudi Arabia	yes	yes
Bolivia	yes	yes	Jamaica	yes	yes	Senegal	yes	yes
Bonaire, Saint Eustatius & Saba	no	yes	Japan	no	no	Serbia	no	yes
Bosnia and Herzegovina	no	yes	Jordan	no	yes	Seychelles	yes	yes
Botswana	no	no	Kazakhstan	no	no	Sierra Leone	yes	yes
Brazil	yes	yes	Kenya	yes	yes	Singapore	yes	yes
Brunei	yes	yes	Kiribati	no	yes	Sint Maarten	no	yes
Bulgaria	no	yes	Kuwait	no	no	Slovakia	no	yes
Burkina Faso	yes	yes	Kyrgyzstan	no	no	Slovenia	no	yes
Burundi	no	yes	Laos	yes	yes	Solomon Islands	yes	yes
Cambodia	yes	yes	Latvia	no	no	Somalia	yes	yes
Cameroon	yes	yes	Lebanon	no	yes	South Africa	no	no
Canada	no	no	Lesotho	no	no	South Korea	no	no
Cape Verde	yes	yes	Liberia	no	yes	South Sudan	yes	yes
Cayman Islands	yes	yes	Libya	no	no	Spain	no	yes

Country	Endemic	Vector presence	Country	Endemic	Vector presence	Country	Endemic	Vector presence
Central African Republic	no	yes	Liechtenstein	no	no	Sri Lanka	yes	yes
Chad	no	yes	Lithuania	no	no	Sudan	yes	yes
Channel Islands	no	no	Luxembourg	no	no	Suriname	yes	yes
Chile	no	no	Macau	yes	yes	Swaziland	no	no
China	yes	yes	Macedonia	no	yes	Sweden	no	no
Christmas Island	no	no	Madagascar	yes	yes	Switzerland	no	yes
Cocos (Keeling) Islands	no	no	Malawi	no	yes	Syria	no	yes
Colombia	yes	yes	Malaysia	yes	yes	Taiwan	yes	yes
Comoros	yes	yes	Maldives	no	yes	Tajikistan	no	no
Congo	no	yes	Mali	yes	yes	Tanzania	no	yes
Cook Islands	no	yes	Malta	no	yes	Thailand	yes	yes
Costa Rica	yes	yes	Marshall Islands	no	yes	The Bahamas	yes	yes
Cote d'Ivoire	yes	yes	Martinique	yes	yes	The Gambia	no	yes
Croatia	no	yes	Mauritania	no	yes	Timor-Leste	yes	yes
Cuba	yes	yes	Mauritius	yes	yes	Togo	no	yes
Curacao	no	yes	Mayotte	yes	yes	Tonga	no	yes
Cyprus	no	no	Mexico	yes	yes	Trinidad and Tobago	yes	yes
Czech Republic	no	yes	Moldova	no	no	Tunisia	no	no
Democratic Republic of the Congo	yes	yes	Monaco	no	yes	Turkey	no	yes
Denmark	no	no	Mongolia	no	no	Turkmenistan	no	no
Djibouti	yes	yes	Montenegro	no	yes	Turks and Caicos Islands	yes	yes
Dominica	yes	yes	Montserrat	yes	yes	Tuvalu	no	yes
Dominican Republic	yes	yes	Morocco	no	no	Uganda	no	yes
Ecuador	yes	yes	Mozambique	yes	yes	Ukraine	no	no
Egypt	no	yes	Myanmar	yes	yes	United Arab Emirates	yes	yes
El Salvador	yes	yes	Namibia	no	yes	United Kingdom	no	no
Equatorial Guinea	yes	yes	Nauru	no	yes	United States	no	yes
Eritrea	yes	yes	Nepal	yes	yes	United States Minor Outlying Islands	no	no
Estonia	no	no	Netherlands	no	yes	Uruguay	no	no
Ethiopia	yes	yes	New Caledonia	yes	yes	Uzbekistan	no	no
Falkland Islands	no	no	New Zealand	no	no	Vanuatu	yes	yes
Federated States of Micronesia	no	yes	Nicaragua	yes	yes	Venezuela	yes	yes
Fiji	no	yes	Niger	no	yes	Vietnam	yes	yes
Finland	no	no	Nigeria	yes	yes	Virgin Islands	yes	yes
France	no	yes	Niue	no	yes	Wallis and Futuna Islands	no	yes
French Guiana	yes	yes	Norfolk Island	no	no	Western Sahara	no	no
French Polynesia	no	yes	North Korea	no	no	Yemen	yes	yes
Gabon	yes	yes	Northern Mariana Islands	no	yes	Zambia	no	yes
Georgia	no	yes	Norway	no	no	Zimbabwe	no	yes
Germany	no	yes	Oman	yes	yes			

Airport ranking

Table D shows the predicted annual number of imported dengue cases for each airport that received at least 10 infections per year in 2011 or 2015.

Table D. Annual estimated imported dengue cases per airport

Code	Imported cases 2011	Imported cases 2015	Name	City	Country/State
MIA	2413	2547	Miami International	Miami	US/Florida
LAX	1518	1871	Los Angeles Intl	Los Angeles	US/California
CDG	941	1227	Charles De Gaulle	Paris-De Gaulle	France/Île-de-France
SFO	831	1166	San Francisco Intl	San Francisco	US/California
MCO	822	1036	Orlando Intl	Orlando	US/Florida
FLL	792	970	Ft Lauderdale Intl	Fort Lauderdale	US/Florida
ORY	652	788	Orly	Paris-Orly	France/Île-de-France
IAH	599	810	George Bush Intercontinental	Houston-Intercontinental	US/Texas
EZE	586	648	Ministro Pistarini	Buenos Aires	Argentina/Autonomous City of Buenos Aires
MAD	385	439	Adolfo Suarez-Barajas	Madrid	Spain/Community of Madrid
DFW	381	478	Dallas/Ft Worth Intl	Dallas/Fort Worth	US/Texas
BNE	379	529	Brisbane Intl	Brisbane	Australia/Queensland
MXP	356	408	Malpensa	Milan-Malpensa	Italy/Lombardy
FCO	343	424	Fiumicino	Rome-Da Vinci	Italy/Lazio
AEP	258	225	Jorge Newbery	Buenos Aires-Newbery	Argentina/Autonomous City of Buenos Aires
MLE	204	214	Ibrahim Nasir International	Male	Maldives/Malé
POS	198	150	Piarco International	Port of Spain	Trinidad and Tobago/Port of Spain
TPA	160	224	Tampa International	Tampa	US/Florida
SXM	159	184	Prinses Juliana International	St. Maarten	Sint Maarten
BCN	148	207	Barcelona	Barcelona	Spain/Catalonia
CUR	147	173	Hato International	Curacao	Curacao
SAN	142	181	San Diego International Airport	San Diego	US/California
HNL	123	153	Honolulu Intl	Honolulu/Oahu	US/Hawaii
BEY	122	162	Rafic Hariri International	Beirut	Lebanon/Beirut
KBL	109	155	Kabul International	Kabul	Afghanistan/Kabul
ACC	97	126	Kotoka International	Accra	Ghana/Greater Accra
SJC	88	99	San Jose Municipal	San Jose	US/California
SAT	80	127	San Antonio Intl	San Antonio	US/Texas
VCE	75	108	Marco Polo	Venice	Italy/Veneto
AUS	71	123	Austin-Bergstrom International Airport	Austin	US/Texas
SMF	70	94	Sacramento International	Sacramento	US/California
OAK	65	65	Metro Oakland Intl	Oakland	US/California
LYS	56	72	Satolas	Lyon	France/Auvergne-Rhône-Alpes
OOL	55	126	Coolangatta	Gold Coast	Australia/Queensland
JAX	53	74	Jacksonville Intl	Jacksonville	US/Florida
MRS	53	69	Marignane	Marseille	France/Provence-Alpes-Côte d'Azur
NCE	53	71	Cote D'Azur	Nice	France/Provence-Alpes-Côte d'Azur
BLQ	49	59	Guglielmo Marconi	Bologna	Italy/Emilia-Romagna
COR	46	78	Pajas Blancas	Córdoba	Argentina/Córdoba
TLS	42	53	Blagnac	Toulouse	France/Occitanie
COO	39	66	Cadjehoun	Cotonou	Benin/Littoral
ONT	39	43	Ontario Intl	Ontario	US/California
SAH	38	11	Sana'a International	Sana'a	Yemen/Sana'a
LIN	38	56	Linate	Milan-Linate	Italy/Lombardy
FAT	37	42	Fresno Yosemite International	Fresno	US/California
SNA	37	58	John Wayne Airport	Orange County	US/California
DLA	33	60	Douala International	Douala	Cameroon/Littoral

Code	Imported cases 2011	Imported cases 2015	Name	City	Country/State
KGL	29	48	Kigali International Airport	Kigali	Rwanda/Kigali
PNS	27	31	Pensacola International	Pensacola	US/Florida
BOD	24	33	Merignac	Bordeaux	France/Nouvelle-Aquitaine
CAY	24	38	Felix Eboue	Cayenne	France/French Guiana
NAN	23	27	Nadi International	Nadi	Fiji/Ba
PBM	22	34	Johan A. Pengel Intl	Paramaribo	Suriname/Paramaribo
CNS	22	37	Cairns International	Cairns	Australia/Queensland
ELP	22	25	El Paso Intl	El Paso	US/Texas
NTE	21	23	Chateau Bougon	Nantes	France/Pays de la Loire
FLR	21	26	Peretola	Florence	Italy/Tuscany
BUR	18	9	Hollywood-Burbank	Burbank	US/California
HOU	18	38	William P Hobby	Houston-Hobby	US/Texas
PPG	17	18	Pago Pago Intl	Pago Pago	American Samoa/Maoputasi County
TRN	17	20	Citta Di Torino	Turin	Italy/Piedmont
ROB	17	17	Roberts Intl	Monrovia-Roberts	Liberia
MFE	15	14	Miller International	McAllen	US/Texas
PBI	15	23	Palm Beach Intl	West Palm Beach	US/Florida
LGB	15	13	Long Beach Municipal	Long Beach	US/California
BZV	15	29	Maya Maya	Brazzaville	Congo/Brazzaville
NAP	14	16	Capodichino	Naples	Italy/Campania
TLH	14	21	Tallahassee International	Tallahassee	US/Florida
MDZ	13	20	El Plumerillo	Mendoza	Argentina/Mendoza
ROS	13	25	Islas Malvinas	Rosario	Argentina/Santa Fe
CRP	13	15	Corpus Christi Intl	Corpus Christi	US/Texas
RSW	13	14	Southwest Florida International	Fort Myers	US/Florida
PMI	13	15	Palma De Mallorca	Palma de Mallorca	Spain/Balearic Islands
MPL	13	18	Mediterranee	Montpellier	France/Occitanie
VPS	12	14	Destin-Ft Walton Beach Airport	Destin-Ft Walton Beach	US/Florida
VRN	12	8	Verona	Verona	Italy/Veneto
FGI	11	20	Fagali'I	Apia	Samoa/Tuamasaga
GNV	11	16	J R Alison Regional Municipal	Gainesville	US/Florida
OGG	11	13	Kahului	Kahului/Maui	US/Hawaii
VLC	11	16	Valencia Airport	Valencia	Spain/Valencian Community
CTA	11	11	Fontanarossa	Catania	Italy/Sicily
BJM	10	17	Bujumbura Intl	Bujumbura	Burundi/Bujumbura Mairie
TAB	10	8	ANR Robinson International	Tobago	Trinidad and Tobago
AGP	10	15	Malaga Airport	Malaga	Spain/Andalusia
ADE	10	3	Aden International	Aden	Yemen/Aden
HRE	10	22	Harare International	Harare	Zimbabwe/Harare
PNR	9	19	Pointe Noire	Pointe Noire	Congo
PUF	9	10	Uzein	Pau	France/Nouvelle-Aquitaine
BIO	9	15	Bilbao Airport	Bilbao	Spain/Basque Autonomous Community
GOA	9	10	Cristoforo Colombo	Genoa	Italy/Liguria
LPA	9	14	Gran Canaria	Gran Canaria	Spain/Canary Islands
GRK	9	11	Regional/R.Gray AAF	Killeen/Fort Hood	US/Texas
WDH	9	10	Windhoek Intl	Windhoek	Namibia/Khomas
MAF	9	13	Midland-Odessa Regl	Midland/Odessa	US/Texas
TRW	9	11	Bonriki International	Tarawa	Kiribati
TSV	9	12	Townsville International	Townsville	Australia/Queensland
PSP	9	11	Palm Springs Muni	Palm Springs	US/California
MLH	9	10	Euroairport	Mulhouse/Basel	France/Grand Est
BES	8	11	Bretagne	Brest	France/Brittany
NKC	8	13	Nouakchott	Nouakchott	Mauritania/Nouakchott
BRC	8	12	San Carlos Bariloche International	San Carlos Bariloche	Argentina/Río Negro
LBB	8	10	Preston Smith Intl	Lubbock	US/Texas
AMA	8	10	Rick Husband Intl	Amarillo	US/Texas
EYW	7	12	Key West Intl	Key West	US/Florida

Code	Imported cases 2011	Imported cases 2015	Name	City	Country/State
NDJ	7	16	Ndjamena	N'Djamena	Chad/N'Djamena
DAL	7	22	Dallas Love Field	Dallas-Love	US/Texas
ECP	5	11	Northwest Florida Beaches International Airport	Panama City	US/Florida
NIM	5	12	Diori Hamani International Airport	Niamey	Niger/Niamey
SPN	3	11	Saipan International	Saipan	Northern Mariana Islands
SFB	0	10	Sanford International	Orlando-Sanford	US/Florida

Table E. The ten routes with the highest predicted number of dengue-infected passengers with final destinations in non-endemic countries with vector presence.

Orig.	Dest.	Pax	Month
SJU (Puerto Rico)	MCO (Florida)	52	Jul
FDF (Martinique)	ORY (France)	34	Aug
CUN (Mexico)	MIA (Florida)	32	Aug
SDQ (Dominican Republic)	MIA (Florida)	30	Aug
CCS (Venezuela)	MIA (Florida)	28	Aug
GDL (Mexico)	LAX (California)	27	Aug
SJU (Puerto Rico)	FLL (Florida)	25	Jul
PUJ (Dominican Republic)	MIA (Florida)	24	Jul
MNL (Philippines)	LAX (California)	23	Jul
SJU (Puerto Rico)	MIA (Florida)	23	Jul

Table F. The ten routes with the highest predicted number of dengue-infected passengers who continue to travel to non-endemic regions. The table lists the direct routes with the highest predicted volume of dengue-infected passengers who continue to travel to non-endemic regions irrespective of vector presence. The last column records the month during which the highest number of infected passengers are predicted.

2011				2015			
Origin	Destination	Pax	Month	Origin	Destination	Pax	Month
BOM	DXB	108	Jul	BOM	DXB	142	Aug
CUN	MEX	86	Aug	DEL	DXB	97	Aug
DPS	PER	77	Jan	CUN	MEX	75	Aug
SDQ	JFK	76	Aug	COK	DXB	72	Aug
STI	JFK	76	Aug	DPS	PER	65	Jan
DEL	DXB	72	Jul	MAA	DXB	59	Aug
MNL	ICN	71	Aug	MNL	ICN	95	Aug
DEL	LHR	65	Aug	HYD	DXB	55	Aug
MNL	NRT	65	Jul	SJU	JFK	57	Aug
MTY	MEX	62	Sep	DEL	LHR	59	Aug

Table G. Yearly and seasonal reporting rates of imported cases in 2011.

	Dec-Feb	Mar-May	Jun-Aug	Sep-Nov	Yearly
Queensland	38.4	25.2	13.7	18.9	24.3
Italy	5.7	4	1.9	7.3	4.4
France	2.2	3.1	4.8	1.3	3
Florida	1.2	0.5	1	2.5	1.3

Supplementary figures

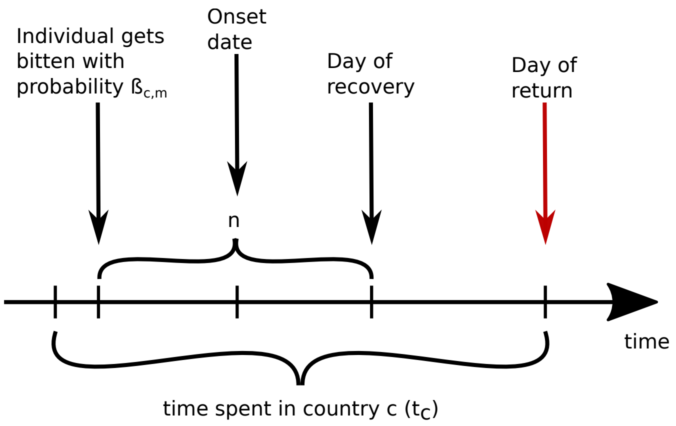

Figure A. Illustration of the possibility of recovery before return. If an individual gets infected with dengue while overseas, but recovers before returning to region r, the individual cannot infect other people in region r.

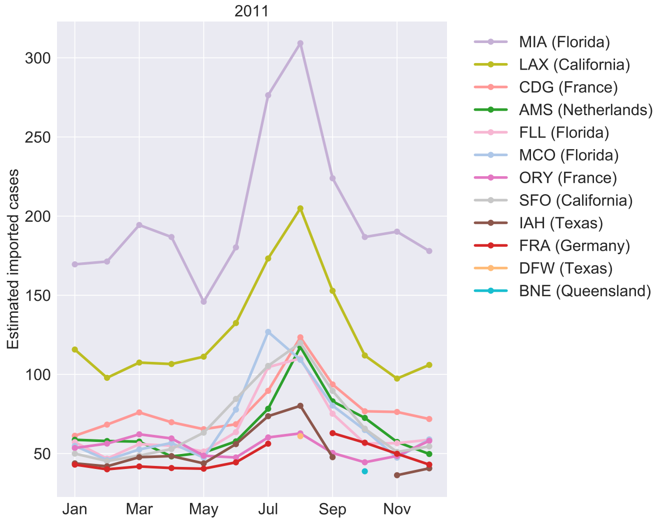

Figure B. Predicted monthly dengue importations by airport for 2011. The number of predicted imported dengue infections for the top ten airports in non-endemic countries/states with vector presence for each month in 2011. A break in a line indicates that the corresponding airport was not amongst the top ten during the respective month. Airports are abbreviated using the corresponding IATA code. A full list of abbreviations can be found in the supplementary material (see Table A)

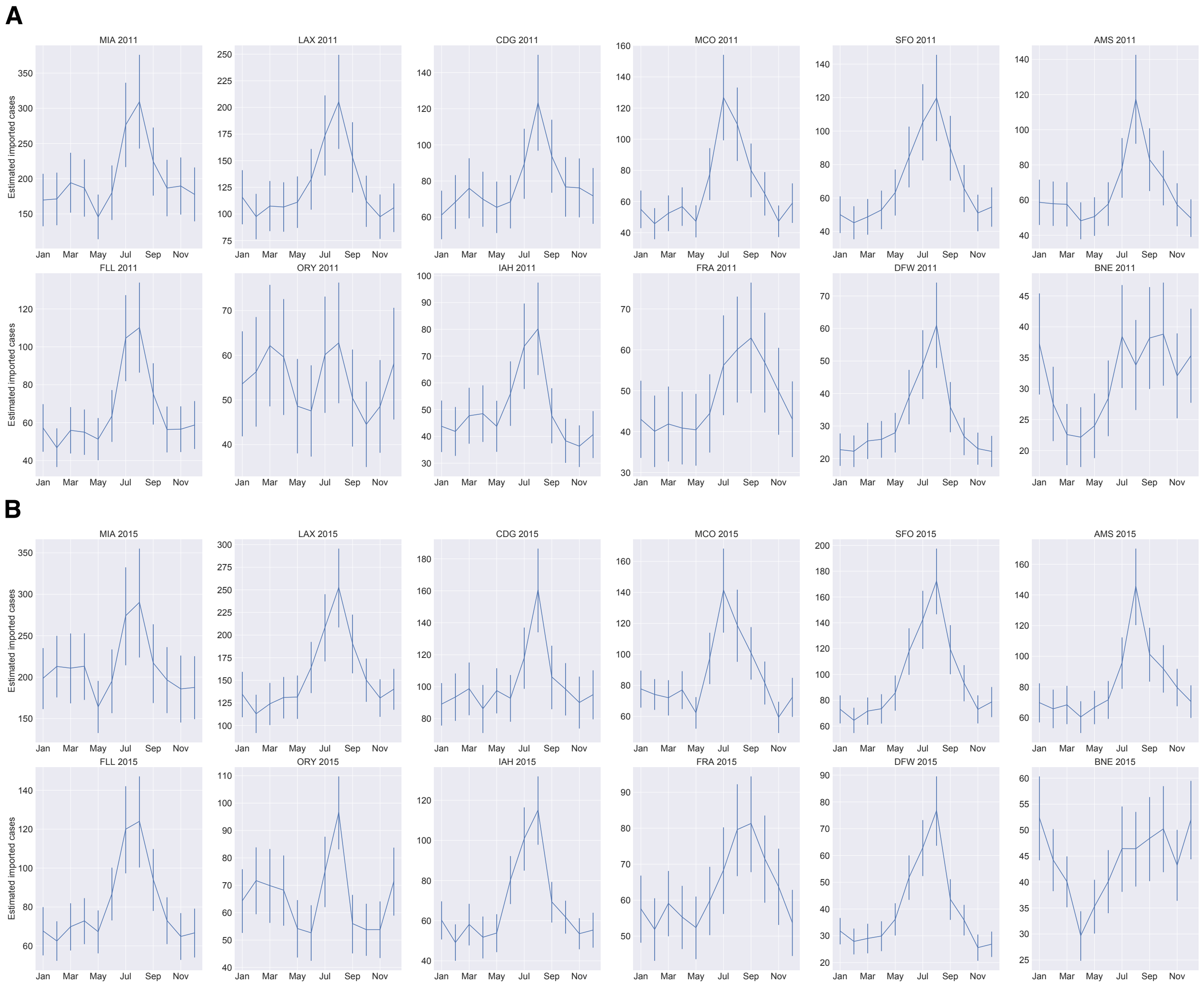

Figure C. Predicted monthly dengue importations by airport The number of predicted imported dengue infections for the top ten airports in non-endemic countries/states with vector presence for each month in (**A**) 2011 and (**B**) 2015. The error bars correspond to ±1 standard deviation. Airports are abbreviated using the corresponding IATA code.

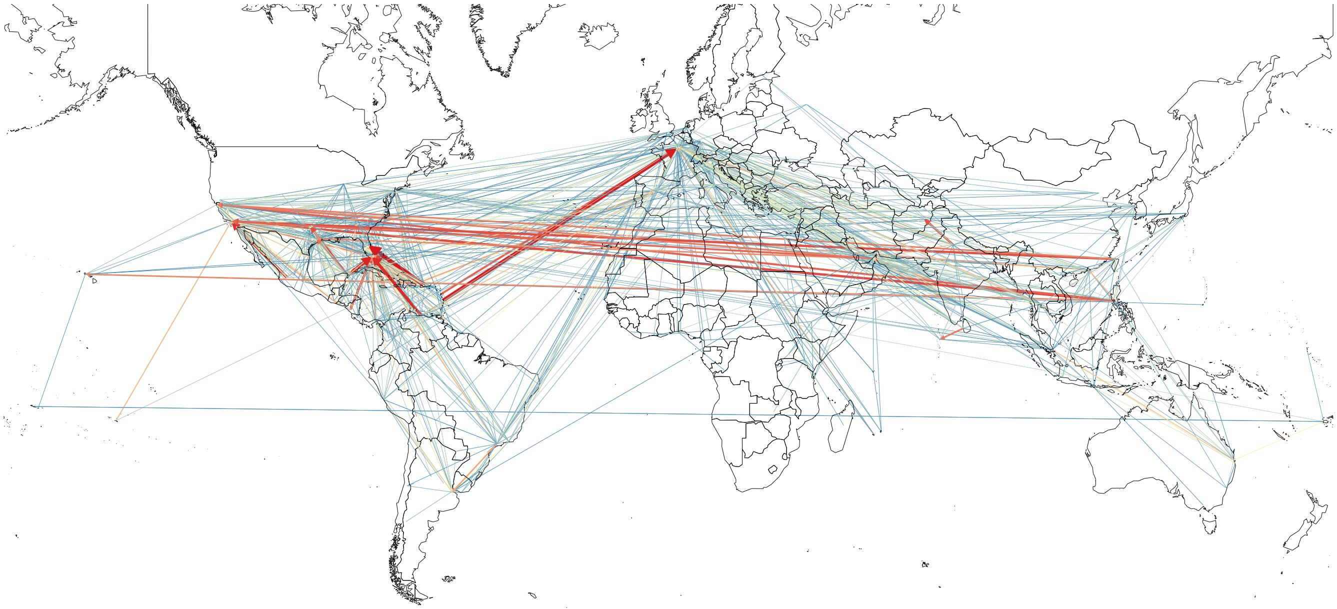

Figure D. Dengue-infected passengers who continue to travel to non-endemic countries/states with vector presence for every route in the air transportation network This map corresponds to August 2015. The thickness as well as the colour of an edge represent the number of infected people travelling along the corresponding route. Blue represents relatively lower numbers of infected people, red represents relatively higher numbers of infected travellers and yellow represents the mid range.

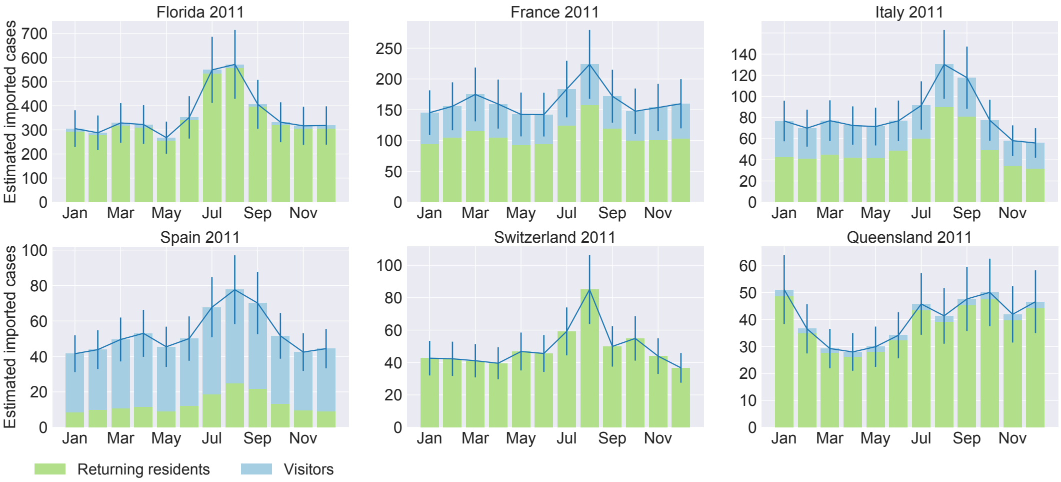

Figure E. Predicted dengue infections imported by returning residents and visitors in 2011 Here we show the results for non-endemic countries/states with vector presence with the highest number of predicted imported dengue cases in 2011. The bars are stacked to distinguish between returning residents (green) and visitors (blue). The blue solid line corresponds to the total number of imported cases. The error bars correspond to the model's coefficient of variation (see Material and methods).

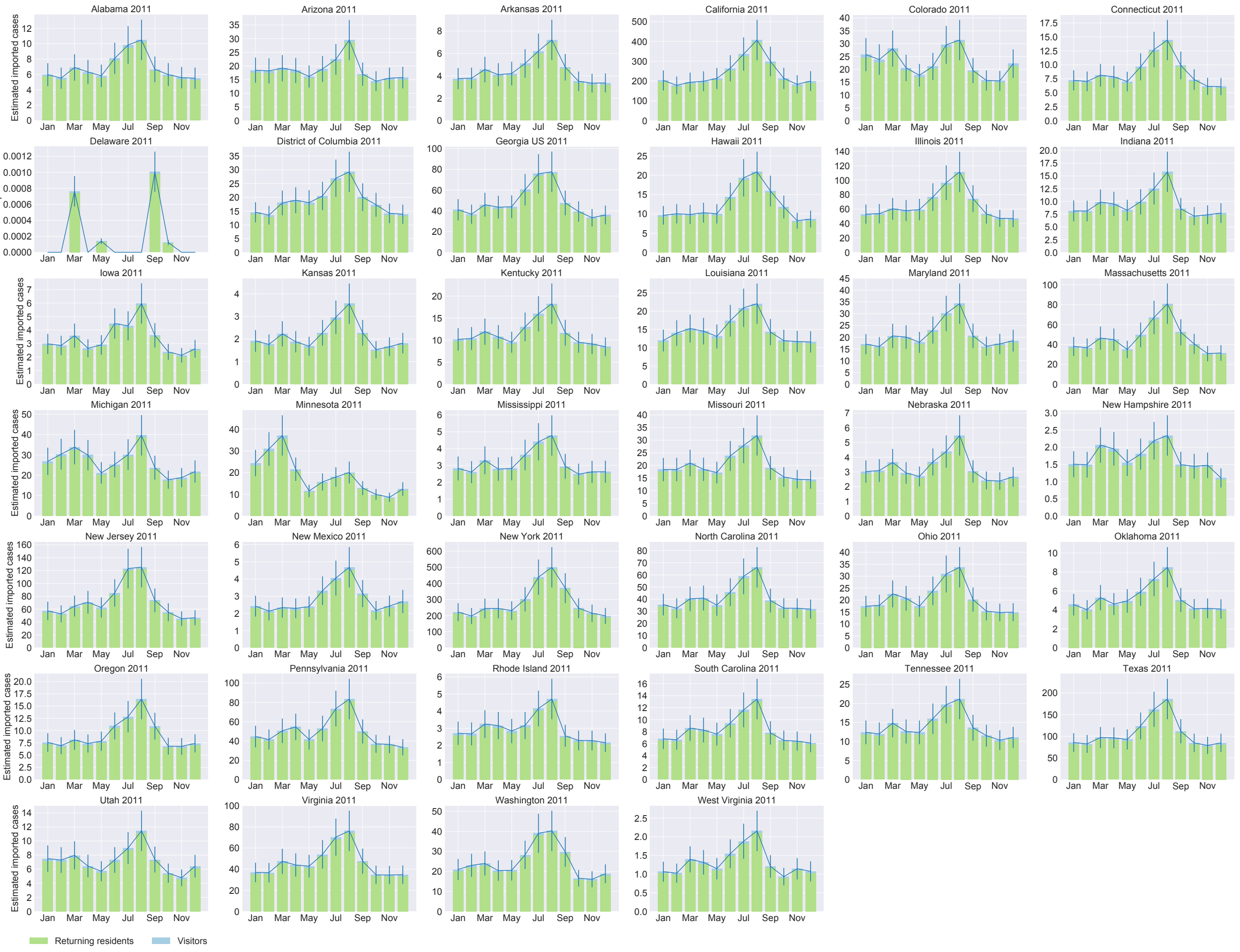

Figure F. Predicted imported dengue infections for returning residents and visitors for US states in 2011. The bars are stacked to distinguish between returning residents (green) and visitors (blue). The blue solid line corresponds to the total number of imported cases. The error bars correspond to the model's coefficient of variation (13.49%) that was inferred through Monte Carlo simulations.

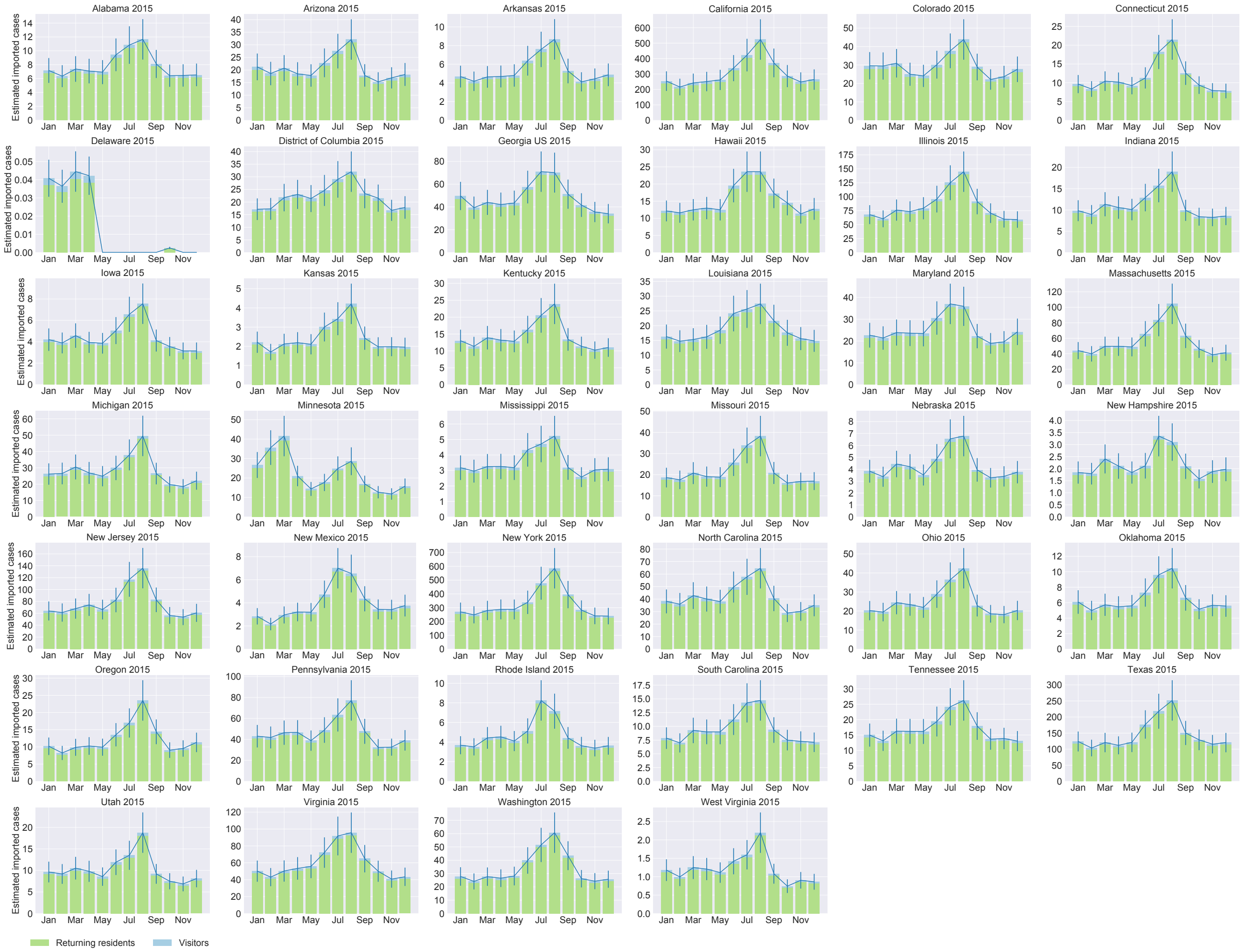

Figure G. Predicted imported dengue infections for returning residents and visitors for US states in 2015. The bars are stacked to distinguish between returning residents (green) and visitors (blue). The blue solid line corresponds to the total number of imported cases. The error bars correspond to the model's coefficient of variation (13.49%) that was inferred through Monte Carlo simulations.

A

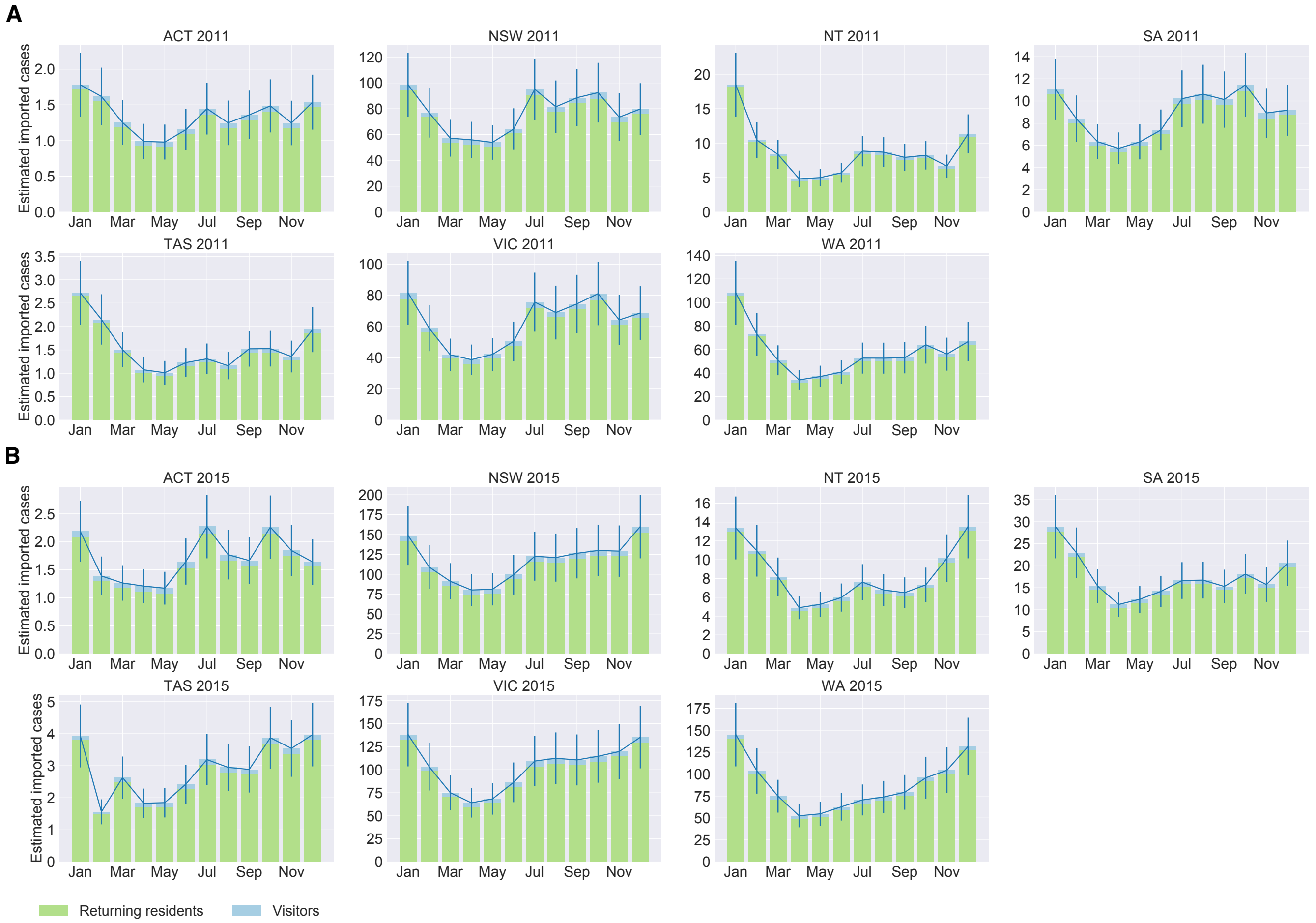

Figure H. Predicted imported dengue infections for returning residents and visitors for Australian states. ACT: Australian Capital Territory, NSW: New South Wales, NT: Northern Territory, SA: South Australia, TAS: Tasmania, VIC: Victoria, WA: Western Australia. The bars are stacked to distinguish between returning residents (green) and visitors (blue). The blue solid line corresponds to the total number of imported cases. The error bars correspond to the model's coefficient of variation (13.49%) that was inferred through Monte Carlo simulations.

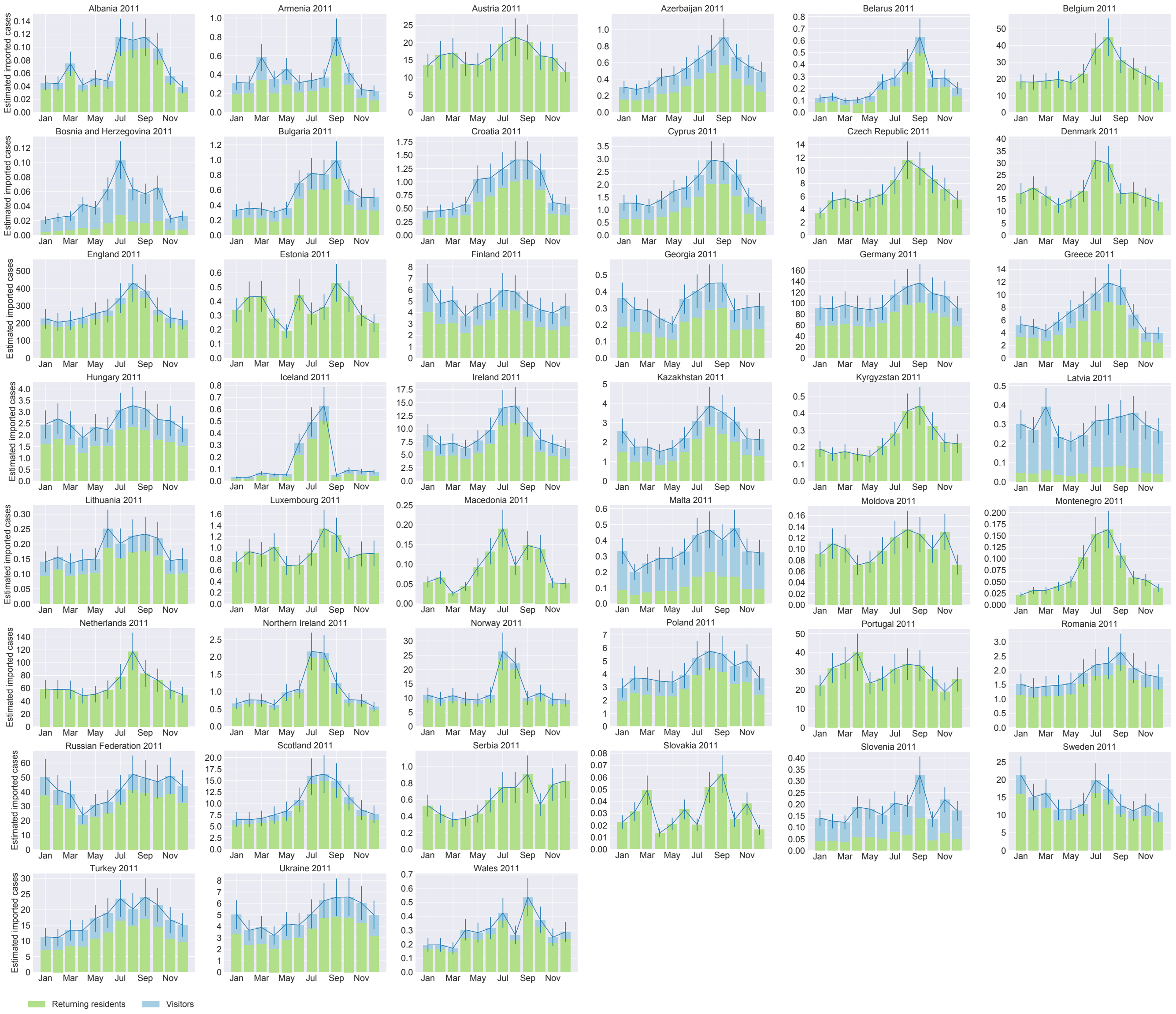

Figure I. Predicted imported dengue infections for returning residents and visitors for European countries in 2011. The bars are stacked to distinguish between returning residents (green) and visitors (blue). The blue solid line corresponds to the total number of imported cases. The error bars correspond to the model's coefficient of variation (13.49%) that was inferred through Monte Carlo simulations.

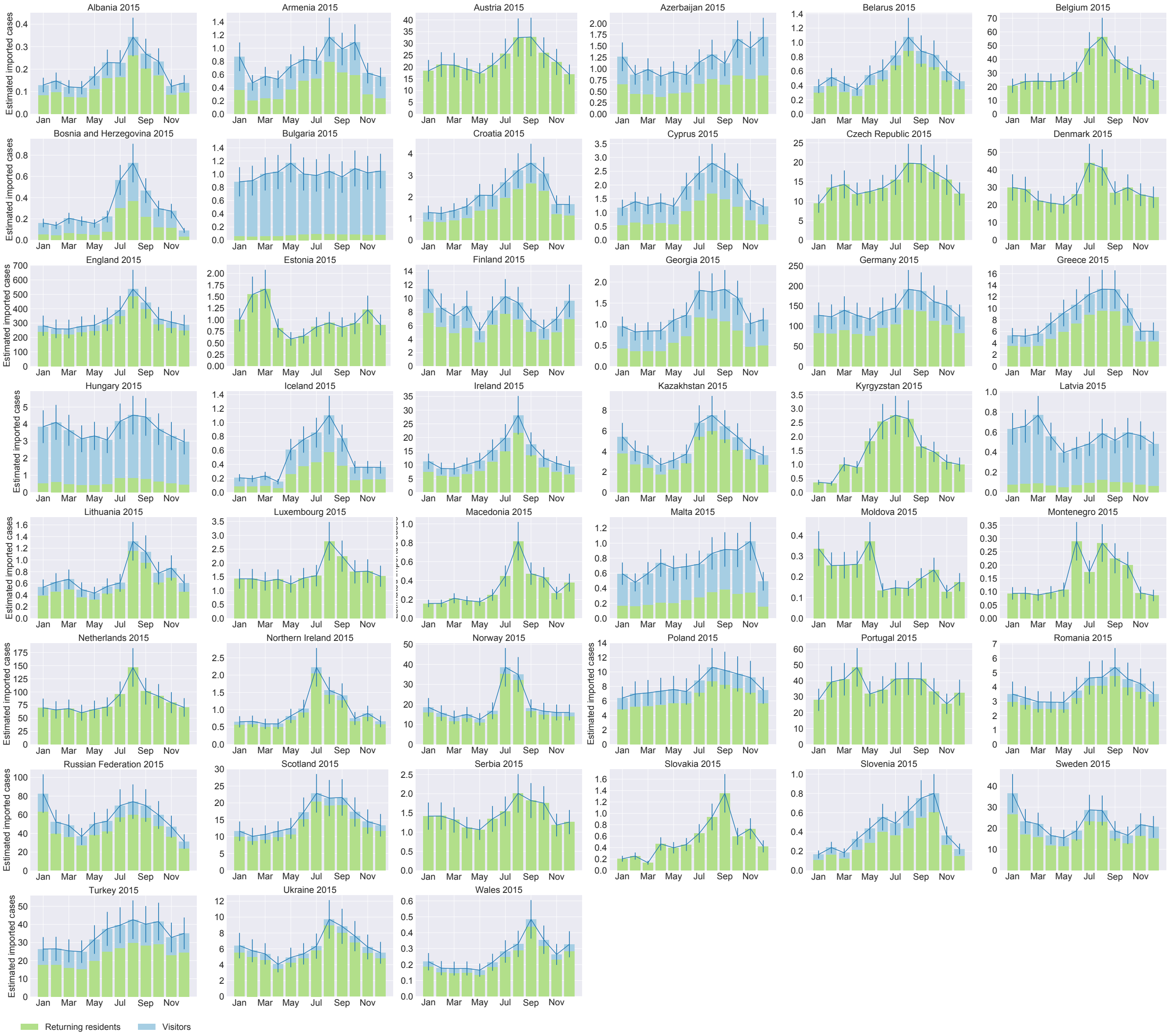

Figure J. Predicted imported dengue infections for returning residents and visitors for European countries in 2015. The bars are stacked to distinguish between returning residents (green) and visitors (blue). The blue solid line corresponds to the total number of imported cases. The error bars correspond to the model's coefficient of variation (13.49%) that was inferred through Monte Carlo simulations.

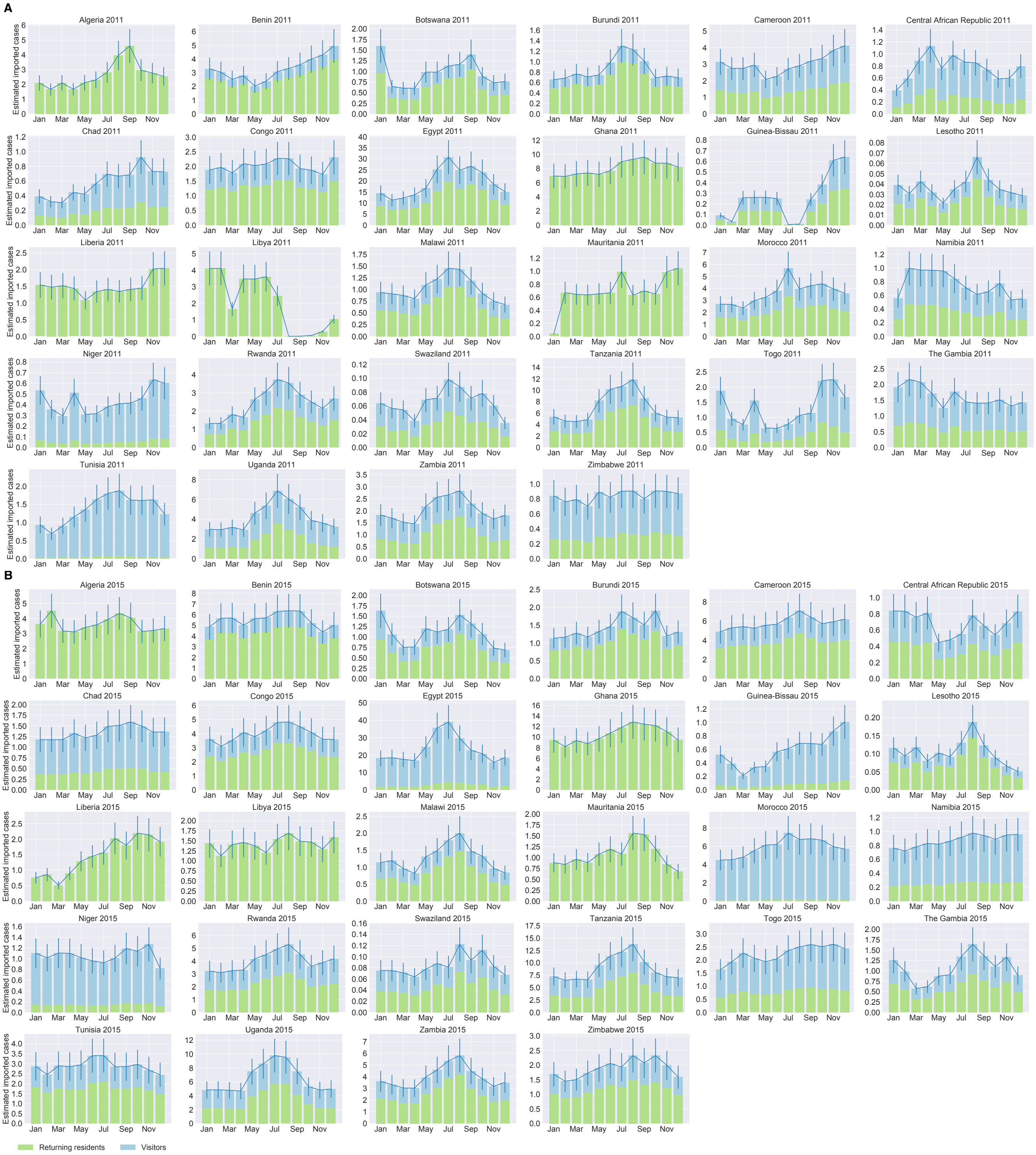

Figure K. Predicted imported dengue infections for returning residents and visitors for non-endemic African countries. The bars are stacked to distinguish between returning residents (green) and visitors (blue). The blue solid line corresponds to the total number of imported cases. The error bars correspond to the model's coefficient of variation (13.49%) that was inferred through Monte Carlo simulations.

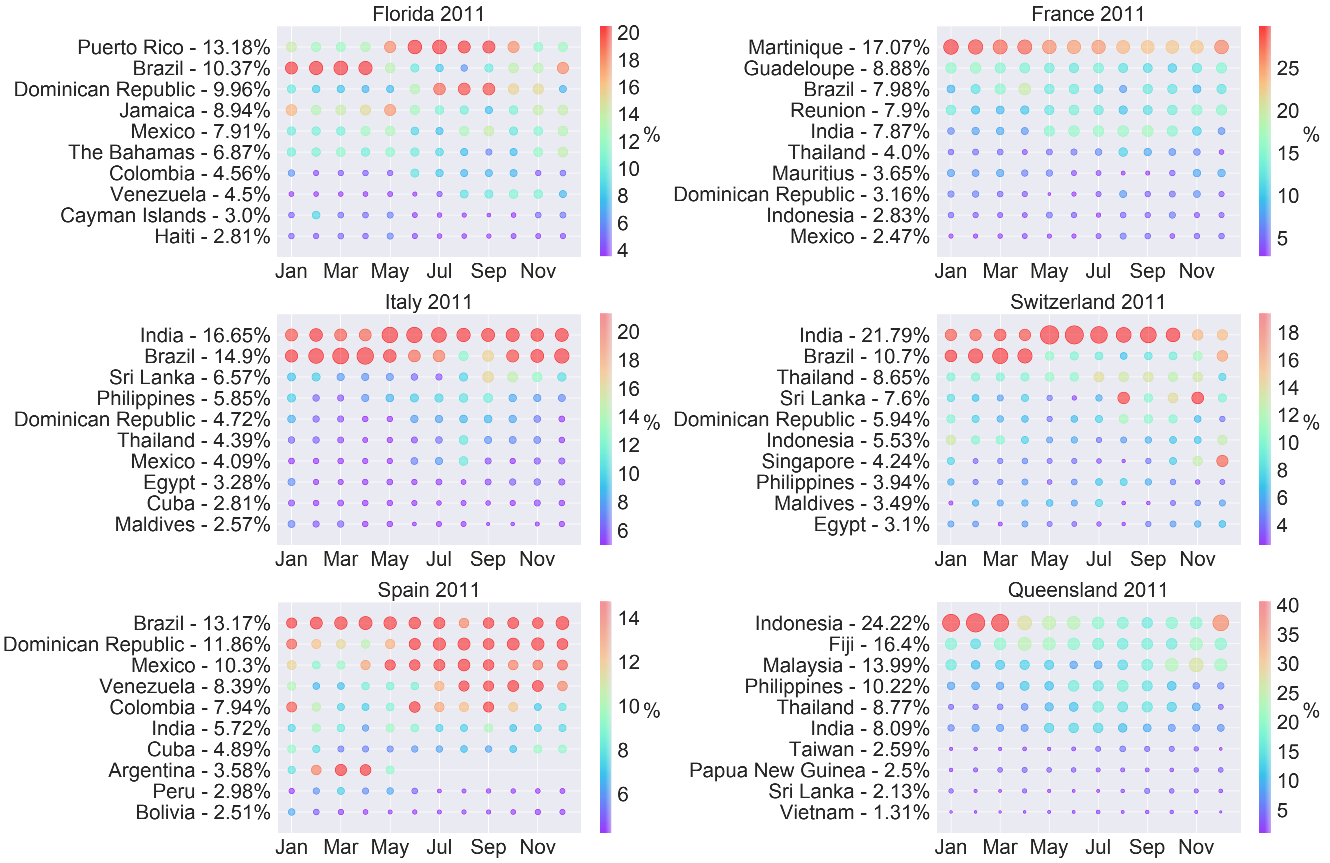

Figure L. Predicted percentage contribution of dengue importations by country of acquisition in 2011. The predicted percentage contribution by source country and month in 2011. The size and colour of the circles indicate the percentage contribution of the corresponding country to the total number of imported cases. The y-labels indicate the yearly percentage contribution of the corresponding source country.

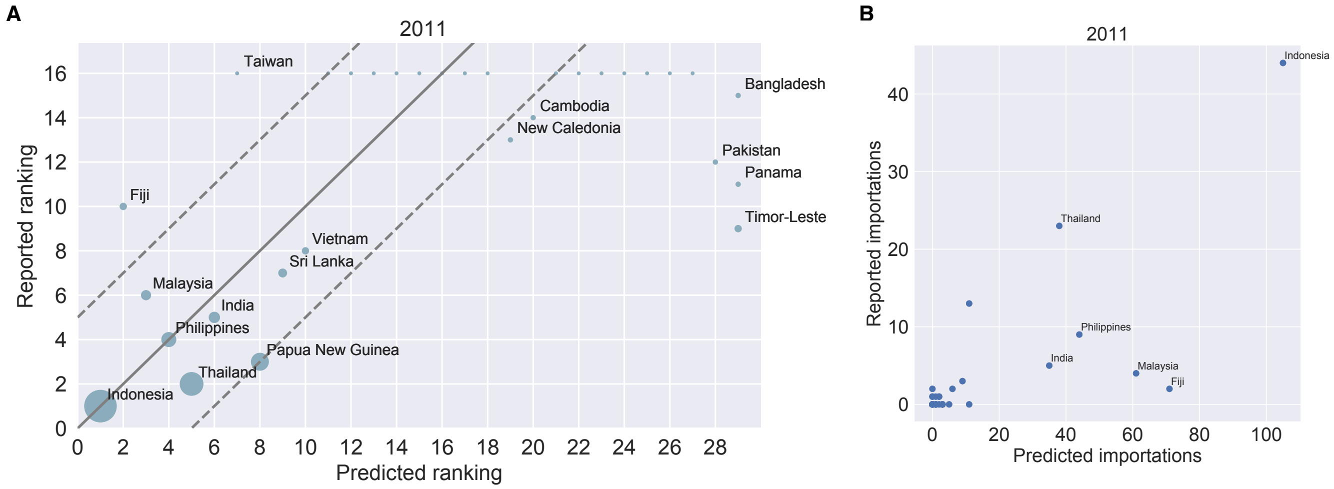

Figure M. Rank-based validation and correlation between reported and predicted imported cases for Queensland in 2011. (**A**) Countries are ranked by the total number of predicted and reported imported dengue cases. The reported ranking is then plotted against the predicted ranking. Countries that were ranked by the model, but did not appear in the dataset receive a rank of $i + 1$, were i is the number of unique importation sources according to the dengue case data. Similarly, countries that appeared in the data and were not ranked by the model receive a rank of $i+1$. For circles that lie on the $x = y$ line (grey solid line) the predicted and reported rankings are equal. Circles that lie between the two dashed lines correspond to countries with a difference in ranking that is less than or equal to five. The circle areas are scaled proportionally to the number of reported cases that were imported from the corresponding country. Spearman's rank correlation coefficient between the absolute numbers of reported and predicted importations is equal to 0.58. (**B**) The absolute number of reported dengue importations are plotted against the absolute number of predicted importations.

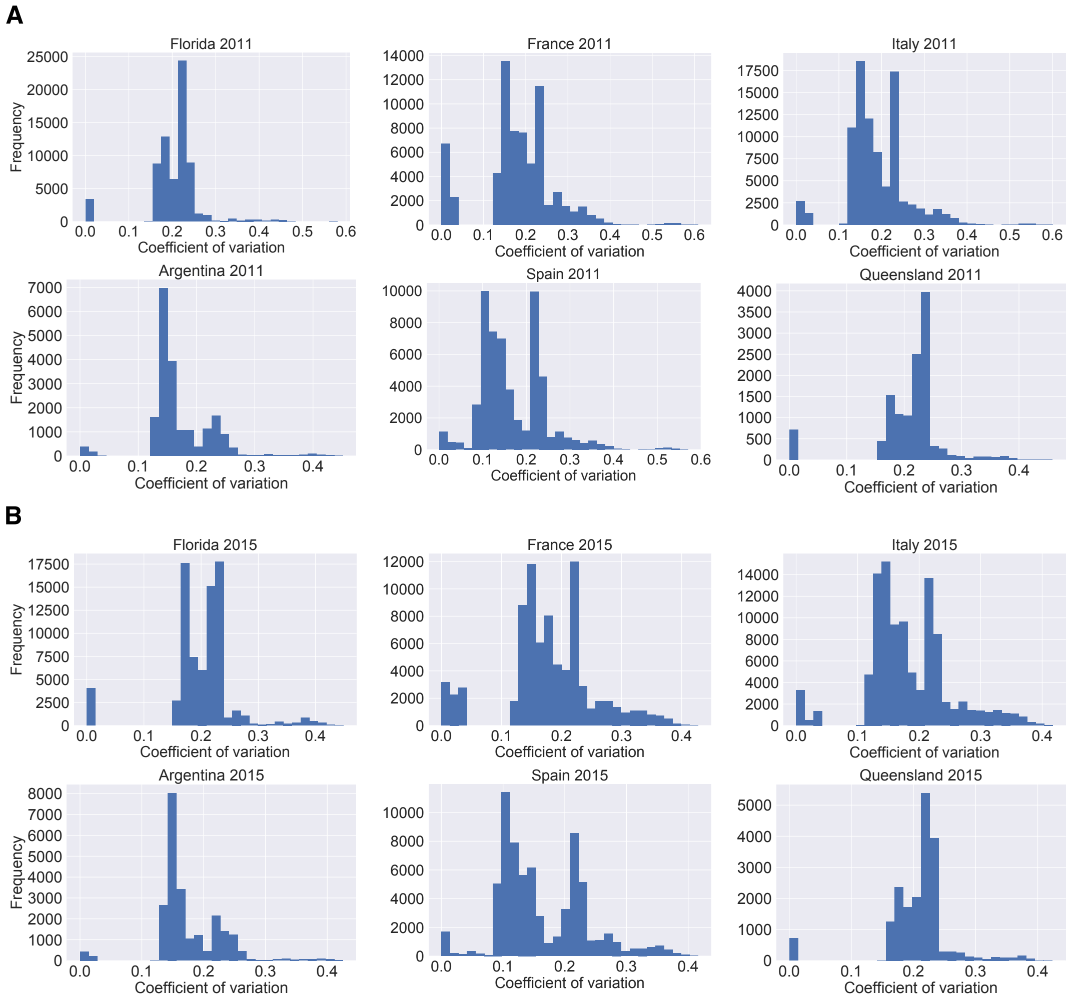

Figure N. The distribution of the coefficient of variation for several destinations. (**A**) Distributions for 2011. (**B**) Distributions for 2015.

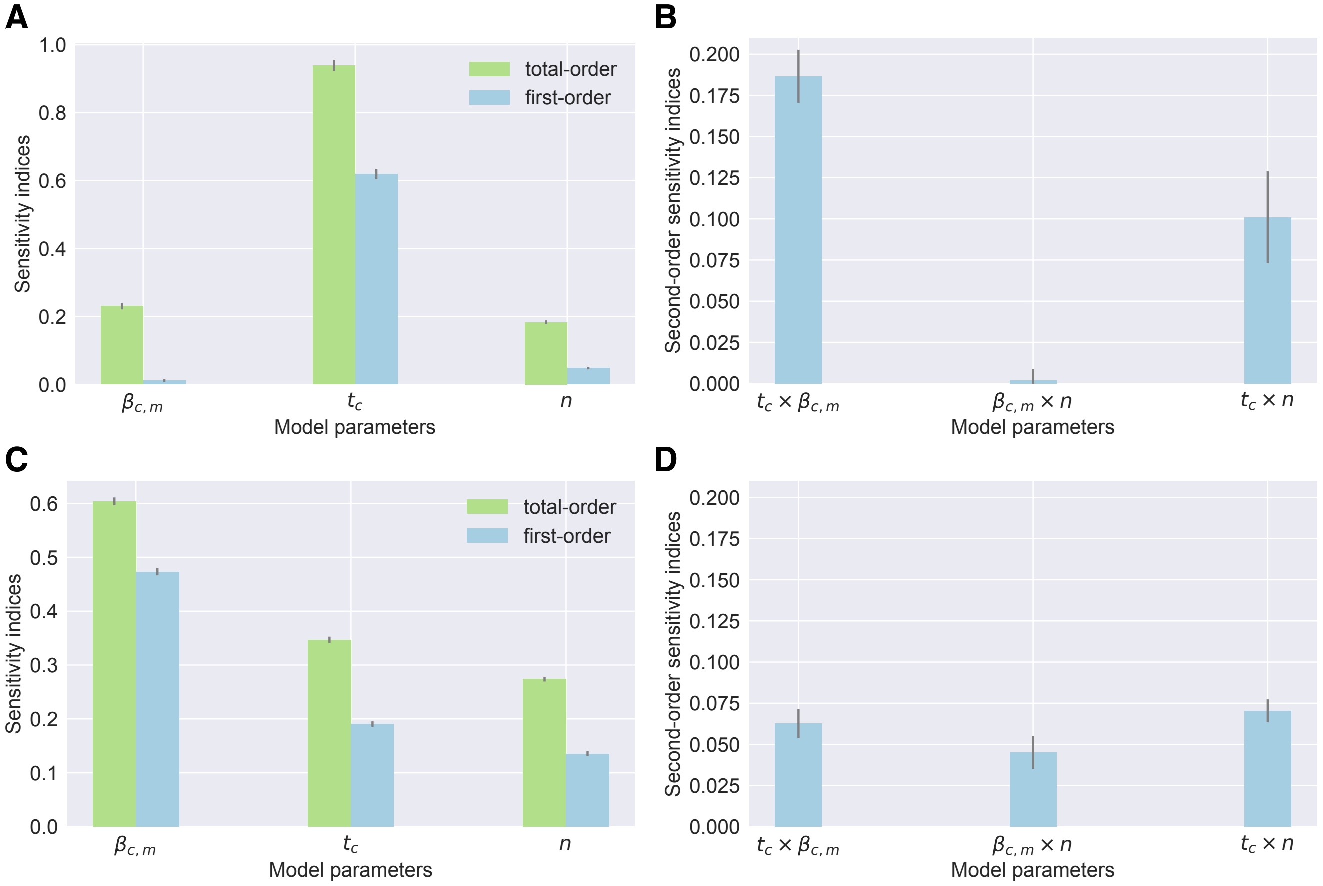

Figure O. Sobol's sensitivity analysis of the model's parameters. Parameter $\beta_{c,m}$ denotes the daily dengue incidence rate of country c during month m, parameter t_c denotes the number of days a traveller who arrives at a given airport has spent in country c and parameter n denotes the sum of the intrinsic incubation period and the infectious period in humans. (**A**) The first-order and total-order indices for the parameter ranges as shown in Table 1 of the main manuscript. The indices indicate that t_c is the most important model parameter. (**B**) The second-order indices for the parameter ranges as shown in Table 1 of the main manuscript. There is significant interactions between parameters t_c and $\beta_{c,m}$ and between parameters t_c and n. (**C**) The first-order and total-order indices for a shorter range of value ([1, 30] days) for parameter t_c. In this case $\beta_{c,m}$ is the most important parameter. (**D**) The second-order indices for a shorter range of value ([1, 30] days) for parameter t_c. There is still significant interaction between parameters t_c and $\beta_{c,m}$ and between parameters t_c and n.

References

[1] Centers for Disease Control and Prevention. 2019 [cited 31/07/19]. Available from: https://www.healthmap.org/dengue/en/.

[2] International Association for Medical Assistence to Travellers (IAMAT). 2018 [cited 6/11/18]. Available from: https://www.iamat.org/.
